# Restricted and repetitive behavior in children with autism during the first three years of life: A systematic review

**DOI:** 10.3389/fpsyg.2022.986876

**Published:** 2022-10-31

**Authors:** Pang Chaxiong, Adele F. Dimian, Jason J. Wolff

**Affiliations:** ^1^Department of Educational Psychology, University of Minnesota, Minneapolis, MN, United States; ^2^Institute on Community Integration, University of Minnesota, Minneapolis, MN, United States

**Keywords:** repetitive behavior, ritualistic, autism spectrum disorder, birth to three, sensory

## Abstract

Restricted and repetitive behavior (RRB) is a core diagnostic feature of autism spectrum disorder (ASD). Previous research shows that RRB is prevalent early in life and observed in neurotypical development as well. Less is known, however, about early RRB patterns, developmental trajectories, and the relation to outcomes for autistic children. The purpose of this systematic review was to synthesize findings from studies examining RRB in autistic children from birth through age 3. A detailed protocol was designed *a priori* based on PRISMA guidelines for systematic reviews. From the published literature, 41 peer reviewed journal articles were identified and included in this review. Our synthesis of the literature suggests that differences in RRB are evident prior to age 2 in children with or who go onto be diagnosed with autism. These differences were evident for both frequency and intensity of RRB across multiple topographies. There were mixed results regarding functional outcomes associated with early RRB, such as cognitive and adaptive behavior, though relations appeared to become stronger as children aged beyond toddlerhood. Notably, level of RRB appears unrelated to autism severity in young autistic children. A wide range of RRB have been reported to be elevated in autistic children during the first years of life, including repetitive motor behaviors, atypical sensory behaviors, insistence on sameness (IS), and self-injurious behaviors (SIBs). In contrast to studies of older children, RRB in very young autistic children do not appear to be associated with functional outcomes but may be valuable to include in early screening efforts.

**Systematic review registration:**

https://osf.io/huzf3, unique identifier: doi: 10.17605/OSF.IO/HUZF3.

## Introduction

Although autism spectrum disorder (ASD) is characterized by both restricted repetitive behaviors (RRB) and social communication characteristics (American Psychiatric Association, 2013), considerably less research has focused on the domain of RRB (Richler et al., 2010; Troyb et al., 2016). What is known about RRB in very early childhood is particularly limited. To date, it is unclear whether and how RRB differentiates autistic children^1^ from neurotypically-developing children (TD) and children with developmental delays (DD). Furthermore, there is limited research tracking the developmental trajectory of RRB and its relations to child outcomes (Leekam et al., 2011). The heterogenous range of RRB topographies and the diverse terminology across research groups, in combination with a lack of universally utilized RRB measures within clinical and service delivery settings, make it difficult to piece together the current state of knowledge on RRB. Gaps in our understanding of RRB hinder the domain's utility to inform identification and intervention efforts in the early years of life when they have the highest potential to capitalize on maximal neural and behavioral plasticity. This review is a step toward filling these gaps by synthesizing the available literature on RRB in autistic children from birth through age 3.

Restricted repetitive behaviors are a class of behaviors characterized by high frequency, repetition, and an insistence or desire for sameness in the environment (Kanner, 1943). In general, RRBs are a continuum of behaviors that may include repetitive motor movements like hand flapping, preoccupation with parts, and circumscribed or restricted interests/routines. While taxonomies of RRB vary (e.g., Lewis and Bodfish, 1998), the Diagnostic and Statistical Manual of Mental Disorders (5^th^ ed.) outlines four patterns of RRB that include (1) repetitive motor, use of objects or speech, or stereotyped behaviors; (2) rigidity including insistence on sameness (IS), ritualized or inflexible adherence to routines; (3) restricted behaviors including circumscribed interests and fixation; and (4) atypical sensory responses including hypo- and hyper-reactivity as well as sensory-seeking behaviors (American Psychiatric Association, 2013). Of the patterns defined, atypical sensory responses and interests is relatively newer to the RRB domain.

The addition of atypical sensory responses to ASD diagnostic criteria has resulted in increased attention to the taxonomy of RRB. Researchers have conducted numerous factor analyses pertaining to RRB presentation within ASD, primarily using caregiver-reported measures. Turner (1999) subdivided the broad range of RRB into two factors: (1) “lower-order” behaviors characterized by repetitive movements including stereotyped motor movements, repetitive manipulation of objects, and repetitive forms of self-injurious behavior (SIB) and (2) “higher order” behaviors including object attachments, IS, repetitive language, and circumscribed interests. Whereas, lower-order repetitive behaviors are characterized by motor rigidity, higher-order repetitive behaviors are considered to be more complex and include cognitive rigidity. Relatedly, RRB topographies have been organized under repetitive sensory motor (RSM) and IS (Bishop et al., 2013; Uljarević et al., 2017; Hiruma et al., 2021). Additional factors, including SIB, have also been identified from validation studies (Lam and Aman, 2007; Mirenda et al., 2010; Bishop et al., 2013) of the Repetitive Behavior Scale-Revised (RBS-R; Bodfish et al., 2000), while a more recent study suggested an additional separate factor for stereotyped speech (Hiruma et al., 2021).

It is currently unclear if and how RRB differentiates autistic children from those who do not have ASD in the first years of life. Restricted and repetitive behavior are not mutually exclusive nor phenomenologically unique to autism. For example, in a review, Leekam et al. (2011) found that all patterns of RRB were also present in children with other disorders (e.g., Tourette syndrome, fragile X, Rett's, and Down's syndromes) as well as in TD children. It is thus necessary to determine if there is sufficient evidence to suggest that aspects of RRB, such as frequency, severity, and developmental trajectory differ between autistic children and TD children and children with DD. The current lack of clarity limits the potential of including aspects of RRB in early screening. This is a critical limitation as accumulating research suggests that RRB may differentiate children who later develop ASD as early as 6–12 months of age (Baranek, 1999; Zwaigenbaum, 2005; Elison et al., 2014; Wolff et al., 2014; Purpura et al., 2017; Miller et al., 2021), perhaps preceding many social communication characteristics which are found to emerge later in toddlerhood (Ozonoff et al., 2010). Furthermore, studies indicate that RRB are reliable predictors of a stable ASD diagnosis between 2 and 9 years of age (Lord and Luyster, 2006; Lord et al., 2006). Closer examination into how RRB looks in autistic children in comparison to TD children and children at elevated likelihood for ASD such as those with DD is needed in order to evaluate the utility of RRB as early markers of ASD.

Another gap in our understanding of RRB is in how it changes across early development in autistic children, as well as how such change compares to children who are TD. There is evidence that RRB in autistic individuals is developmentally dynamic (Leekam et al., 2011; Berry et al., 2018). A key complication is that RRB are also common in TD children during early childhood and similarly undergo rapid change through early school age. Some topographies of RRB, such as motor stereotypies, may peak in the first year of life and gradually decrease thereafter (Thelen, 1979; MacLean et al., 1991; Sifre et al., 2021). Other RRB, such as IS and behavioral rigidity, are often observed from toddlerhood through preschool age, for example, when it is common for young children to request the same song, movie, or clothing repeatedly, to carry around a certain item, or to insist that a task be completed in a specific order (Evans et al., 1997; Zohar and Felz, 2001). An understanding of how RRB associated with ASD changes over early childhood, and in relation to TD, has the potential to inform developmental mechanisms as well as timing of intervention when warranted.

Relatedly, relations of RRB to other developmental outcomes such as social-communication, cognitive ability, and adaptive functioning are also unclear. This knowledge is critical in decisions to intervene for RRB (i.e., some forms of RRB may presage outcomes that warrant prevention, while others may be innocuous or even beneficial). In comparison to social communication characteristics, much more controversy surrounds the intervention of RRB, namely in regards to if and what specific RRB require intervention. On the one hand, RRB can scaffold the learning of more complex skills. For example, motor stereotypies may be foundational to the acquisition of goal-directed behavior, while IS or ritualistic behavior may provide stability and order to a preschooler's erratic and increasingly demanding environment. Relatedly, autistic adults report that RRB are functional and adaptive, enhancing their abilities to function, control stimulation, and cope with stress (Manor-Binyamini and Schreiber-Divon, 2019). Indiscriminant prevention or suppression of RRB has the potential for both short- and long-term adverse effects. Conversely, RRB also have the potential to interfere with learning and the acquisition of skills if they serve as barriers to opportunities and experiences necessary for healthy development. For example, socialization may become limited if a child is engaging in high or interfering levels of RRB (Nadig et al., 2010). Furthermore, parents of autistic children report that RRB is one of the most challenging features associated with ASD (South et al., 2005; Raulston and Machalicek, 2018) and indicate high levels of stress associated with RRB (Hayes and Watson, 2013). Given these complex, yet critical perspectives, it is important to clarify what is known about early RRB in relation to key functional outcomes.

In summary, limited knowledge of RRB in very early childhood hinders the utility of RRB as a core feature of ASD in efforts to further refine early identification and intervention. With increasing prevalence estimates of ASD, recent clinical advancements in diagnosing ASD at a younger age, and thus ability to provide intervention services earlier, it is critical to clarify what is currently known of the distinctiveness of RRB in young children with ASD. Thus, the purpose of this review was to synthesize findings from studies examining RRB in autistic children from birth through age 3. Our review addresses three questions: (1) What is known about the types of RRB that differentiate autistic children from TD children and children with DD?; (2) How do the frequency, the duration, and the severity of RRB in early child development change over time in children with ASD?; and (3) What is known about RRB in relation to child outcomes for autistic children birth through age 3?

## Materials and methods

Our study protocol was drafted using an earlier version of Van den Akker et al. (2020), a product that was a result of the Society for Improving Psychological Science. The protocol is registered with the Open Science Framework on May 22^nd^, 2022 (https://osf.io/huzf3).

### Study selection

This review included peer-reviewed, quantitative studies that examined RRB in autistic children birth through age 3 (0 to < 48 months). Studies that included children older than 48 months were eligible for inclusion if the mean age of autistic participants was under 48 months or if data on autistic participants under 48 months could be extracted. Studies that did not include autistic children were excluded. Qualitative, intervention, non-human animal studies, gray literature, book chapters, reviews, and studies written in a language other than English were also excluded. Studies that fit our inclusion criteria but did not contain data contributing to at least one research question were also excluded (e.g., Ramsey et al., 2018; Zwaigenbaum et al., 2018; Stephenson et al., 2021).

**Figure 1:**
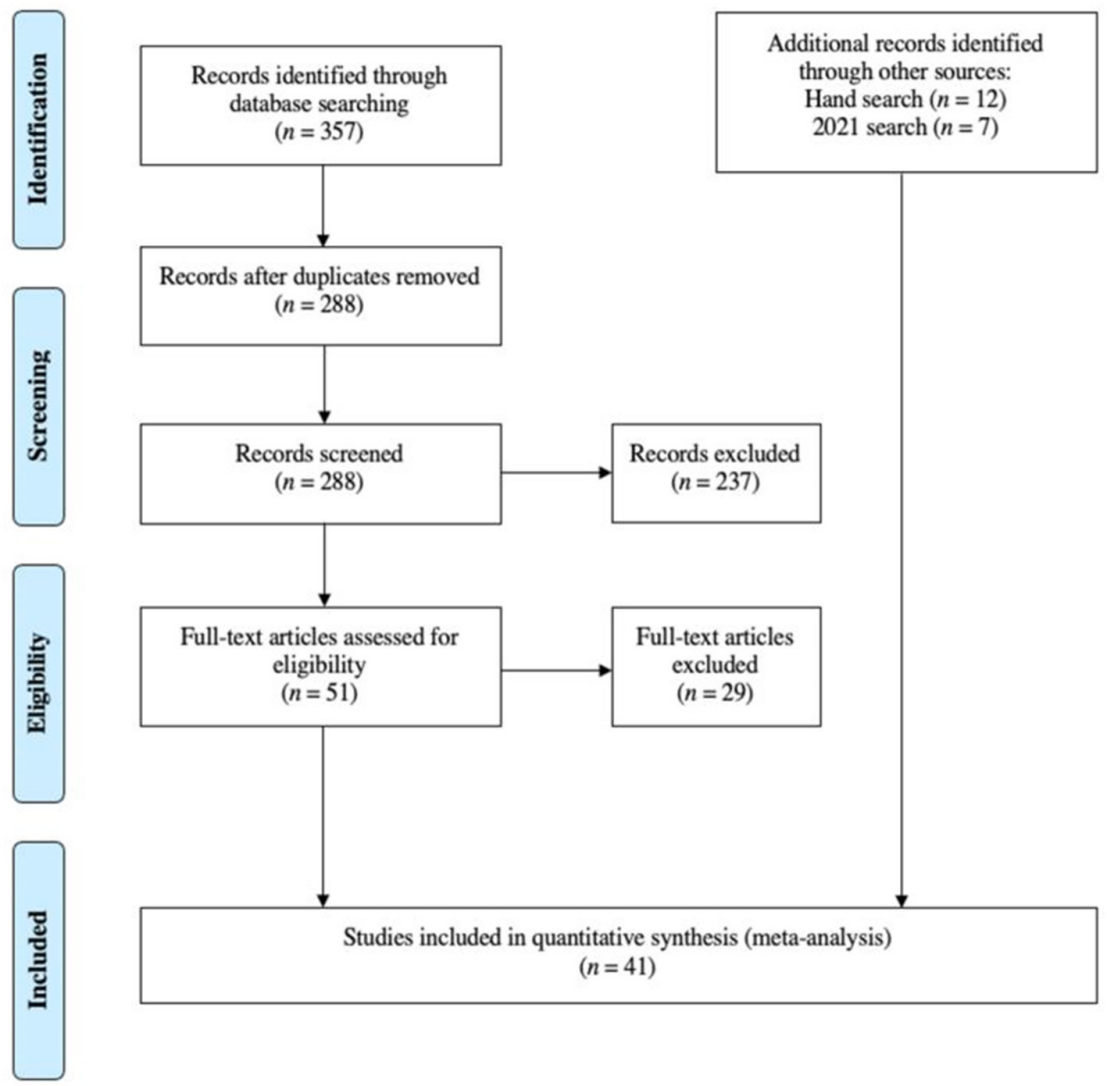
PRISMA flow chart.

Eligible studies were located through a search of PubMed, PsycINFO, Academic Search Premier, Education Source, and Education Resources Information Center (ERIC) using the following keywords and roots: (autis^*^ *or asd*) AND (*restricted repetitive behavio*r^*^ OR *rrb*) AND (early develop^*^ OR earlychild^*^ OR *birth to three*). This search procedure was originally conducted in September of 2019 and identified 357 studies. After duplicates were removed, 288 studies were screened by title and abstract following the inclusion criteria above, identifying 51 studies for full text review. Twenty-two studies met eligibility criteria. A hand search of the references of these studies identified an additional 12 studies for a total of 34 studies. A second search using the same procedure was conducted in April of 2021, identifying 7 additional studies for a total of 41 studies included in this review (see Figure 1). Studies identified through this process represent literature published between 1999 and 2020. Study selection was independently conducted by the first two authors on Rayyan, a free web application that allows for blind coding of studies for systematic reviews (Ouzzani et al., 2016). All inter-rater discrepancies were resolved by consensus.

### Data collection

A coding rubric was jointly developed by the three authors and was iteratively updated throughout the data collection process. Extracted data included (a) study descriptives (i.e., authors, date of publication); (b) study methodology [i.e., study purpose, RRB measures used (direct or indirect)]; (c) sample characteristics [i.e., age, sex, race/ethnicity, diagnosis, higher-familial-likelihood sample (infant siblings of an autistic child), prospective sample, group matching]; and (d) findings pertaining to research questions. To enable uniform reporting of sample characteristics across studies, the research team calculated descriptive statistics (i.e., sample size, age mean, and percentages of female/male/racial/ethnic groups) as needed based on the information provided.

The first author coded all 41 studies while the second author independently coded 37% (*n* = 15) of the studies. Periodic consensus meetings were held throughout data collection to discuss inter-rater discrepancies. Interrater agreement was calculated by [agreements/(agreements + disagreements)] ^*^ 100. Mean IRA was 94% (range 80–100%).

### Data analysis

RQ1 was addressed using studies that compared RRB in autistic children to TD children and children with DD (*n* = 26). RQ2 was addressed using studies with longitudinal or repeated measures analyses that examined changes in dimension(s) of RRB over a minimum of two time points (*n* = 24). Lastly, RQ3 was addressed using studies that examined RRB in relation to concurrent and/or distal child outcomes (*n* = 24).

#### RRB categories

Given the heterogeneity of RRB topographies, a lack of uniformity in the terminology and measures used across research groups, we synthesized research using five broad categories of RRB:

- *Overall-RRB:* Findings pertaining to global RRB measures and scores that reflect topographies from different patterns of RRB.
- *Repetitive sensory-motor behavior (RSM):* Findings pertaining to stereotyped movements and preoccupations with a specific interest, activity, or object.
- *Insistence on sameness (IS):* Includes findings pertaining to compulsive, ritualistic behaviors, and resistance to changes in routines.
- *Self-injurious behavior (SIB):* Includes findings pertaining to repetitive behaviors that cause or have the potential to cause bodily injury ranging from minor to severe.
- *Atypical sensory behaviors:* Includes findings pertaining to hyper- or hypo-activity to sensory input and sensory-seeking behaviors. Findings here are limited to studies that used a sensory-specific measure.

Table 1 presents the measures reflected in the literature. We organized findings according to these RRB categories for several reasons. Repetitive sensory-motor behavior and Insistence on sameness are included as previous studies have indicated that the heterogeneous range of RRB topographies load most consistently into these two factors (Bishop et al., 2013; Uljarević et al., 2017; Hiruma et al., 2021). Self-injurious behavior is also included as factor analyses of the RBS-R—a parent-report measure frequently used in our included studies—report this as a third and separate factor from RSM and IS (Lam and Aman, 2007; Mirenda et al., 2010; Bishop et al., 2013). Finally, sensory behaviors is included as this topography has been added to the RRB domain as part of the diagnostic criteria for ASD (American Psychiatric Association, 2013). We reasoned that organizing findings according to this categorization would facilitate cross-study synthesis.

**Table 1:** Measures used for defining RRB categories and outcomes.

**Measures**	
**RRB categories**	
Overall-RRB	ADI-R RRB algorithm scores, ADOS RB algorithm scores, BISCUIT RRB subscale scores, DBC-P RRB scores, SRS RRB scores, SORF RRB scores, RBS-R total scores
Repetitive sensory motor	RBS-R stereotyped subscale scores, RBS-R repetitive subscale scores, RMRIS composite scores, RSMS composite scores, measures and study specific codes that use RRB with object and RRB with body cluster scores, ADI-R items: repetitive use of objects, complex mannerisms, hand/finger mannerisms, unusual preoccupations, and unusual attachments, ADOS items: hand/finger mannerisms, complex mannerisms, repetitive behaviors, stereotyped language, SORF items: repetitive speech/intonation, repetitive use of objects, repetitive body movements, excessive interest in particular objects, actions, or activities, clutches particular objects, sticky attention to objects, fixation on parts of objects, lack of playing with a variety of toys conventionally
Insistence on sameness	RBS-R compulsive subscale scores, RBS-R restricted subscale scores, ADI-R items: difficulties with changes in routine, compulsions/rituals, resistance to trivial changes in the environment, ADOS items: adherence to routines/rituals, SORF items: ritualized patterns of behavior and marked distress over change
Self-injurious behavior	RBS-R self-injurious subscale scores, ADI-R and ADOS items: presence of self-injurious behavior
Atypical sensory behaviors	ITSP, SEQ, SPA, SSP, ADI-R items: unusual sensory interests, abnormal/idiosyncratic response to sensory stimuli, sensitivity to noise, ADOS items: presence of sensory interests, SORF items: adverse response to sensory stimuli and unusual sensory exploration/interest
**Child outcomes**	
ASD severity	ADI-R total scores, ADOS total scores, CARS total scores
RRB severity	ADI-R RRB algorithm scores, ADOS RRB algorithm scores, SRS RRB scores, RBS-R total scores, study-specific observational RRB codes
Social-communication/ interaction	ADI-R social and communication scores, ADOS social affect scores, CSBS social scores, CSBS speech scores, MSEL expressive language scores, MSEL receptive language scores, MSEL visual reception scores, PEP-R developmental age, PLS-4 auditory comprehension scores, PLS-4 expressive communication scores, VABS communication domain scores, VABS socialization domain scores
Cognitive Ability	CSBS symbolic scores, DAS-II verbal reasoning scores, DAS-II non-verbal reasoning scores, M-P-R developmental index, MSEL ELC scores, MSEL NVDQ scores, MSEL VDQ scores, WAIS-IV scores, WISC-IV scores
Adaptive function	VABS ABC scores, VABS daily living skills scores
Motor	MSEL gross motor scores, VABS motor scores
Self-regulation	ADOS aggressive-disruptive scores, CBCL dysregulated profile scores, CBCL internalizing scores, CBCL externalizing scores, CBCL pervasive developmental problems scores, CBCL total problems scores

ADI-R, Autism Diagnostic Interview-Revised; ADOS, Autism Diagnostic Observation Schedule; BISCUIT, Baby and Infant Scale for Children with aUtIsm Traits; CARS, Childhood Autism Rating Scale; CBCL, Child Behavior Checklist; CSBS, Communication and Symbolic Behavior Scales Developmental Profile; DAS-II, Differential Ability Scales; DBC-P, Developmental Behavior Checklist-Primary Carer Version; DPA, Developmental Play Assessment; ITSP, Infant/Toddler Sensory Profile; M-P-R, Merrill-Palmer-Revised Scales of Development; MSEL, Mullen Scales of Early Learning; PEP-R, Psychoeducational Profile-Revised; PLS-4, Preschool Language Scale Fourth Edition; RBS-R, Repetitive Behavior Scale-Revised; RSMS, Repetitive and Stereotyped Movement Scales; SEQ, Sensory Experiences Questionnaire; SORF, Systematic Observation of Red Flags for Autism Spectrum Disorders in Young Children; SPA, Sensory Processing Assessment; SSP, Short Sensory Profile; VABS, Vineland Adaptive Behavior Scales; WAIS-IV, Wechsler Adult Intelligence Scale; WISC-IV, Wechsler Intelligence Scale for Children.

#### Group definitions

To address RQ1, we defined ASD, TD, and DD groups as the following:

- *ASD Group:* Children who are identified as having ASD, autism, autistic disorder, Aperger' syndrome, PDD-NOS, and higher-familial-likelihood children with ASD (HL-ASD). If a study differentiated between these diagnoses (e.g., compared autistic children to children with PDD-NOS), these groups were combined to create one ASD group with aggregated results.
- *TD Group:* Children who are identified as typically-developing, children with no known disabilities or concerns, and lower-familial-likelihood without ASD (LL-Neg) and higher-familial-likelihood children without ASD (HL-Neg).
- *DD Group:* Children who are identified with a global developmental, speech/language, or motor delay/difficulties or children with a non-spectrum condition such as a learning or intellectual disability.

#### Dimensions of RRB change

To address RQ2, we examined changes over time in the following RRB dimensions:

- *Severity:* Changes in overall/composite RRB scores.
- *Frequency*: Changes in the number of times a participant/group engages in a specific RRB topography/category.
- *Duration:* Changes in the length of time a participant/group engages in a specific RRB topography/category.

#### Early child outcomes

To address RQ3, we presented findings on relations between the five RRB categories described above and concurrent or distal measures of the following child outcomes: ASD severity, RRB severity, social-communication/interaction (including expressive and receptive communication), cognitive ability, adaptive function, motor, and self-regulation. Table 1 presents the measures that examined each outcome. Restricted and repetitive behavior was included as an outcome to evaluate associations between categories of RRB both concurrently and over time (e.g., whether early RRB predicts later RRB). Outcomes were not specified a priori; all relations between RRB categories and outcomes that emerged from the reviewed studies are reported.

## Results

### Study characteristics

The description of studies included in this review are presented in Table 2. Hereafter, studies are referred to as S*n*, where *n* refers to the order a study appears in Table 2. Altogether, the studies in our sample included a total of 5,816 participants. Sample sizes ranged from *N* = 20 (S26) to *N* = 760 (S22). Male participants made up the majority of study samples (i.e., ranging from 50.0% in S11, S14, and S20 to 92.3% in S5). Twenty-six (63.4%) studies reported some racial/ethnic information, indicating majority-White samples (i.e., ranging from 39.7% in S14 to 92.5% in S8). Nineteen (46.3%) studies used prospective ASD samples and seven (17.1%) studies used higher-familial-likelihood samples. Among the 30 studies that compared ASD samples to TD and/or DD samples, seven (23.3%) studies matched participants by chronological age and six (20.0%) studies matched participants by maturational age. Twenty-two (53.7%) studies used direct measures of RRB, 12 (29.3%) studies used indirect measures, while seven (17.1%) studies used a combination of both.

**Table 2:** Study characteristics.

**Study description**			**Participants**				
**Study**	**Aim/Relevant research question(s)**	**RRB measure**	**Overall: *N* *F* (%) *M* (%)**	**ASD *n*^CA/MA, HL, P^ Age (m) Age Range**	**TD *n* Age (m) Age Range**	**DD *n* Age (m) Age Range**	**Race/ethnicity (%): 1. Asian 2. Black 3. Hispanic/Latino 4. White 5. Other 6.Not reported**
1. Baranek (1999)	“...Explored the usefulness of sensory-motor measures in addition to social behaviors as early predictors of autism during infancy”	Study-specific observational RRB codes	32 40.6 59.4	11^CA^ 63.0 –	11 53.0 –	10 65.0 –	1.6.3 2. 3.1 3. 3.1 4. 84.4 5. 3.1 6.–
2. Baranek et al. (2007)	“..Examine the prevalence and nature of hyperresponsiveness to sensory stimuli in children with autism compared to children with other developmental delays and children developing typically.”	SPA	139 26.6 73.4	56 43.8 5.0–83.0^O^	53 29.4 5.0–83.0^O^	30 38.7 5–83.0^O^	1. – 2. 18.7 3. 3.6 4. 64.7 5. 5.8 6. 7.2
3. Baranek et al. (2006)	“...Describes a new caregiver-report assessment, the Sensory Experiences Questionnaire (SEQ), and explicates the nature of sensory patterns of hyper- and hyporesponsiveness, their prevalence, and developmental correlates in autism relative to comparison groups”	SEQ	258 33.7 66.3	80* 39.8 23.0–80.0	110 29.3 5.0–49.0	68 33.6 11.0–64.0	1. – 2. 14.3 3. 1.2 4. 48.8 5. 4.3 6. 31.4
4. Barber et al. (2012)	“...Extend Watt's (2008) analysis by examining the frequency, repertoire, and duration of RSB demonstrated by the same sample of 50 children with ASD compared to 50 children with TD matched on developmental level rather than chronological age”	Observational RRB codes from Watt et al. (2008)	100 14.0** 86.0**	50^MA, P^ 21.3 18.0–26.9	50 14.6 8.3–24.6	–	1.4.0** 2. 16.0** 3. 8.0** 4. 72.0** 5. – 6.–
5. Ben-Itzchak and Zachor (2020)	“...Examined the developmental changes over time of adolescents diagnosed in toddlerhood with autism spectrum disorder and searched for child characteristics at toddlerhood that predict outcome at adolescence”	ADOS; SRS-2	65 7.7 92.3	65 26.4 15.6–37.2	–	–	–
6. Bruckner and Yoder (2007)	“...The object play of 27 young children with autism was measured in a semi-structured context to quantify restricted object use”	ADOS; DPA	27 14.0 86.0	27 33.6 –	–	–	1. – 2. 17.0 3. – 4. 65.0 5. – 6. 17.0
7. Cox et al. (1999)	“...The association between, and stability of clinical diagnosis and diagnosis derived from the Autism Diagnostic Interview-Revised...was examined in a sample of prospectively identified children with childhood autism and other pervasive developmental disorders assessed at age of 20 and 42 months”	ADI-R	45 22.2 77.8	21^P*^ 21.0 –	15 20.3 –	9 20.7 –	–
8. Damiano et al. (2013)	“...Determine the extent to which RSMs are elevated in Sibs-ASD with and without later diagnoses of ASD, relative to Sibs-TD, after matching on mental age”	RSMS	40 32.5 67.5	8^CA, MA, HL, P^ 15.0 12.0–23.0	32^ 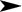 ^ 15.2 12.0–23.0	–	1. – 2. – 3. – 4. 92.5 5. – 6. 7.5
9. Dow et al. (2019)	“...Examine the utility of the Systematic Observation of Red Flags...as a level 2 screener for autism spectrum disorder (ASD) in toddlers during a naturalistic video-recorded home observation”	ADOS; SORF	228 27.2 72.8	84^P^ 20.7 –	62 20.4 –	82 20.3 –	1.2.2 2. 14.0 3. 11.0 4. 71.1 5. 11.4 6.–
10. Elison et al. (2014)	“...Characterize patterns of RRBs representative of either a disorder-specific deficit or a pattern representing familial liability for ASD in a large sample of 12-month-olds”	ADOS; RSMS	158 39.9 60.1	30^HL, P^ 12.7 11.7–15.5	128^ 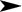 ^ 12.8 11.5–5.1	–	–
11. Germani et al. (2014)	“..Assessed sensory processing differ- ences between 24-month infants at high-risk of autism spectrum disorder (ASD), each with an older sibling with ASD, and low-risk infants with no family history of ASD”	ITSP	90 50.0 50.0	14^HL, P^ 24.7^O^ –	76^ 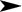 ^ 24.7^O^ –	–	–
12. Guthrie et al. (2013)	“...Examine the (a) short-term stability of clinical diagnoses, (b) concurrent and predictive utility of ADOS-T classifications and scores, and (c) short-term change in symptom severity”	ADOS	82 22.0 78.0	56^P^ 19.1 –	26 20.1 –	–	1.2.4 2. 14.6 3. 9.8 4. 73.2 5. 9.8 6. –
13. Grzadzinski et al. (2020)	“...Examined sensory reactivity patterns at 14 months, changes from 14 to 23 months, and later ASD severity at 3–5 years of age in children (*n* = 87) at elevated likelihood of ASD”	SEQ; SPA	87 31.0 69.0	87 14.0 –	–	–	1. – 2. 19.5 3. – 4. 69.0 5. 10.3 6. 1.1
14. Harrop et al. (2015)	“Determine if the associations between developmental variables (non-verbal and verbal) and chronological age and RRBs differ by gender”	ADOS; Observational RRB codes from Harrop et al. (2014)	58 50.0 50.0	58 37.3 –	–	–	1.24.1 2. 6.9 3. 12.1 4. 39.7 5. 17.2 6. –
15. Harrop et al. (2014)	“...Investigate the presence of ‘lower order' RRBs in children in the toddler to preschool period (2–5 years at entry) in two groups of children (ASD and TD)”	Study-specific observational RRB codes	93 12.9 87.1	49^MA^ 45.5 –	44 24.2 –	–	1. – 2. – 3. – 4. 89.2 5. – 6. 10.8
16. Honey et al. (2008)	“...Examine repetitive behaviors in a large cohort of 2–4 year old children with ASD and speech and language delays, and track developmental changes over a 13 month period”	ADI-R	104 20.2 79.8	79* 37.1^O^ 24.0–48.0^O^	–	25 37.1^O^ 24.0–48.0^O^	–
17. Jacques et al. (2018)	“...investigated repetitive behaviors and object explorations in 49 autistic and 43 age-matched typical young children (20–69 months)”	Study-specific observational RRB codes	92 22.8 77.2	49^CA^ 47.1 20.0–69.0^O^	43 42.8 20.0–69.0^O^	–	–
18. Kim and Lord (2010)	“Restricted and repetitive behaviors (RRBs) observed during the Autism Diagnostic Observation Schedule...were examined in a longitudinal data set of 455 toddlers and preschoolers...with clinical diagnosis of Autism Spectrum Disorders..., a non-spectrum disorder..., or typical development....”	ADOS	455 23.7 76.3	192* 28.4^O^ 8.0–56.0^O^	173 28.4^O^ 8.0–56.0^O^	90 28.4^O^ 8.0–56.0^O^	1.1.0 2. 20.0 3. – 4. 73.0 5. 4.0 6. 2.0
19. Lawson et al. (2018)	“...Examine gender differences in early autism manifestations and cognitive development in a community-ascertained sample of children with ASD from 24 to 48 months of age”	ADOS	67 31.3 68.7	67^P^ 25.7 23.0–33.0	–	–	–
20. Loh et al. (2007)	“...Describe the quality and quantity of stereotyped and repetitive motor stereotypies and posturing in an initial sample of ‘baby sibs' subsequently diagnosed with ASD, undiagnosed siblings, and low-risk comparison infants, highlighting differences between these groups at 12 and 18 months”	Study-specific observational RRB codes	32 50.0 50.0	8^HL, P^ 12.1 –	24^ 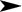 ^ 12.5 –	–	–
21. MacDonald et al. (2007)	“...Compare levels of vocal and motor stereotypy in 2-, 3-, and 4-year-old children with autism or PDD-NOS and typically developing age-matched children”	Study-specific observational RRB codes	60 – –	30^CA^ 41.3 24.0–59.0	30 41.7 27.0–59.0	–	–
22. Matson et al. (2009)	“...Determine the nature and rate of stereotypies and ritualistic behaviors as measured by a recently developed scale for infants with ASD”	BISCUIT	760 30.4 69.6	261* 26.6^O^ 17.0–37.0^O^	499 26.6^O^ 17.0–37.0^O^	–	1. – 2. 35.9 3. 2.4 4. 50.1 5. – 6. 3.6
23. Mirenda et al. (2010)	“...Examine external validity...of the RBS-R by examining correlations between RBS-R factors and several variables that have been related to repetitive behavior in previous research”	ADI-R; ADOS; RBS-R	287 15.7 84.3	287 40.7 24.1–64.0	–	–	1. – 2. – 3. – 4. 71.8 5. – 6. 28.2
24. Mooney et al. (2006)	“...Examined whether repetitive behaviors are a feature of autism in children aged less than 51 months, independent of developmental level, by investigating the relationship between chronological age, developmental age and the presence of repetitive behaviors”	ADI-R; DBC-P	55 16.4 83.6	40 36.8 22.0–51.0^O^	–	15 37.5 22.0–51.0^O^	–
25. Mooney et al. (2009)	“...Aimed to use factor analysis to investigate the existence of a higher- and lower-level repetitive behavior categorization of ADI-R repetitive behavior items in young children diagnosed with a PDD”	ADI-R	198 16.7 83.3	137 38.1 20.0–55.0	–	61 39.8 22.0–55.0	–
26. Moore and Goodson (2003)	“...Twenty children who presented with severe interactional and communication difficulties at age 2 underwent a comprehensive assessment for autism, and were reassessed at age 4–5”	ADI-R	20 20.0 80.0	19^P*^ 34.0^O^ 29.0–42.0^O^	–	1 34.0^O^ 29.0–42.0^O^	–
27. Morgan et al. (2008)	“...Examine group differences and relationships with later developmental level and autism symptoms using a new clinical tool developed to measure repetitive and stereotyped movements (RSM) in young children”	RSMS	125 16.0 84.0	50^CA − TD, MA − DD, P^ 21.3 –	50 21.1 –	25 20.6 –	1.2.4 2. 16.0 3. 5.6 4. 76.0 5. – 6.–
28. Nilsson Jobs et al. (2019)	“...Assessed preschool staff's ratings of social communication and interaction (SCI) and restricted and repetitive behaviors (RRBs) in 3-year-old siblings of children with ASD, either diagnosed (*n* = 12) or not diagnosed (*n* = 36) with ASD, and typically developing siblings with no family history of ASD (*n* = 16)”	ADOS; RBS-R	64 51.6 48.4	12 37.7 –	52^ 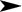 ^ 36.9 –	–	–
29. Ozonoff et al. (2008)	“...Examine whether the manner in which infants explore objects at 12 months of age differs in children later diagnosed with autism or ASD”	ADOS; Study-specific observational RRB codes	66 38.0 62.0	9^HL, P^ 12.0 –	47 12.1 –	10 12.2 –	–
30. Ray-Subramanian and Ellis Weismer (2012)	“...Examined whether language skills and non-verbal cognitive skills were associated with clinician-observed restricted and repetitive behaviors (RRBs) in a sample of 115 children with autism spectrum disorders (ASD) at ages 2 and 3”	ADOS	115 15.7 84.3	115* 31.0 –	–	–	1. – 2. 1.7 3. 2.6 4. 86.1 5. 9.6 6. –
31. Richler et al. (2007)	“Restricted and repetitive behaviors (RRBs)...were examined in 165 children with Autism Spectrum Disorders (ASD), 49 children with non-spectrum developmental disorders (DD), and 65 children with typical development (TD) at approximately 2 years of age”	ADI-R	279 23.7 76.3	165^P^ – –	65 – –	49 – –	1. – 2. 30.5 3. – 4. 65.2 5. – 6. 3.9
32. Richler et al. (2010)	“...Using longitudinal data, collected when children were approximately 2, 3, 5, and 9 years of age, to investigate how RRBs change in children with ASD over time, and to identify the variables that predict these changes”	ADI-R	214 19.6 80.4	161^P*^ 28.9^O^ –	–	53 28.9^O^ –	1. – 2. 31.3 3. – 4. 66.8 5. – 6. 1.9
33. Rogers et al. (2003)	1. “...Examine parental reports of sensory symptoms in young children with several different developmental disabilities including autism, in order to examine the presence and specificity of these symptoms in early autism” 2. “...Examine relationships of sensory symptoms with intellectual ability, age, overall severity of autism, and severity of specific symptom clusters associated with autism” 3. “...Evaluate the relative contribution of sensory symptoms to the acquisition of adaptive behavior in young children”	ADI-R; ADOS; SSP	102 – –	26^MA − TD^ 33.7 26.0–41.0	24 19.5 12.0–35.0	32 33.2 24.0–47.0	–
34. Schertz et al. (2016)	1. “...Are there associations among RBS-R total and subscale scores and concurrent measures of cognition, adaptive behavior, and social competence?” 2. “...Are there differences in the RBS-R total and subscale scores for younger and older toddler groups and how do any age-delineated toddler differences compare to subscale scores previously reported for older age groupings?”	ADOS; RBS-R	143 18.0 82.0	143 25.5 16.0–31.0	–	–	1.5.0 2. 13.0 3. 9.0 4. 62.0 5. 10.0 6.–
35. Stronach and Wetherby (2014)	“...What is the relationship between RRB in clinic and home observations compared to (a) concurrent measures of social communication and (b) later measure of cognitive development and autism symptoms?”	ADOS; RMRIS	55 21.8 78.2	55^P^ 19.8 –	–	–	1.1.8 2. 10.9 3. 12.7 4. 76.4 5. 10.9 6.–
36. Troyb et al. (2016)	1. “...To help clarify the relationship between RRBs and later functioning, the current study used a longitudinal design, multiple measures of RRBs, and comprehensive assessment at outcome” 2.“...Determine how well RRBs add to previously identified predictors of outcome”	Aggregated review of ADI-R, ADOS, and clinical judgment	40 15.0 85.0	40 26.2 19.3–33.7	–	–	1.5.0 2. 5.0 3. – 4. 90.0 5. – 6.–
37. Uljarević et al. (2018)	“..Examined the link between poor self-regulation...and core autism symptoms, as well as with developmental level, in a sample of 107 children with autism spectrum disorder (ASD) aged 19–46 months”	ADOS	107 16.8 83.2	107 31.6 19.0–46.6	–	–	1.0.9 2. 13.1 3. 4.7 4. 71.0 5. 6.5 6. 3.7
38. Watt et al. (2008)	1. “...Describe the duration, frequency, and types of RSB displayed by children with ASD, DD, and TD between 18 and 24 months of age during systematic observation and to examine group differences” 2. “...Examine concurrent and predictive relationships between RSB in the second year and developmental level...and adaptive behavior…” 3. “...Examine concurrent and predictive relationships between RSB in the second year and social symptoms in the second and fourth years”	ADOS; study-specific observational RRB codes	125 16.0 84.0	50^CA − TD, MA − DD, P^ 21.3 18.2–26.9^O^	50 21.1 18.2–26.9^O^	25 20.6 18.2–26.9^O^	1.2.4 2. 16.0 3. 5.6 4. 76.0 5. – 6. –
39. Wetherby et al. (2004)	“...Determining which red flags distinguished children with and without ASD and the accuracy of identification of ASD based on the red flags”	SORF	54 13.0 87.0	18^CA − TD, P^ 21.0 13.1–26.9	18 20.3 13.6–24.4	18 18.4 13.0–24.9	1. – 2. – 3. 3.7 4. 85.2 5. – 6. 14.8
40. Wolff et al. (2014)	“...Clarify the development of repetitive behavior from 12 to 24 months among TD children and high-risk siblings who did and did not meet criteria for ASD at 2 years of age. We were particularly interested in examining how subtypes of RRBs manifest during this early interval”	ADOS; RBS-R	229 39.3 60.7	36^HL, P^ 12.6 –	193^ 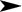 ^ 12.6 –	–	1.2.0 2. 2.4 3. 4.4 4. 84.8 5. 6.4 6.–
41. Wolff et al. (2019)	“...Characterize early patterns of sensory responsiveness in high-risk toddlers who did and did not go on to meet diagnostic criteria for ASD”	RBS-R; SEQ	466 39.1 60.9	74^HL, P^ 12.7 –	392^ 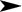 ^ 12.6 –	–	1. – 2.– 3. 6.4 4. 88.4 5. 7.5

Only data on participants birth to < 48 months of age are presented. For retrospective and longitudinal studies, only data at intake/Time 1 are presented. Restricted and repetitive behavior subtypes investigated: IS, Insistence on sameness; RSM, Repetitive sensory motor [includes repetitive and stereotyped behaviors (RSB)]; SIB, Self injurious behavior; Sensory, hyper/hypo-responsive, sensory-seeking, Global, all four areas. Subgroups: ASD, Autism spectrum disorder; DD, Developmental delay/disabilities; TD, Typically developing. Subgroup characteristics: ^P^, Prospective sample; ^HL^, Higher-familial-likelihood sample; ^CA^, ASD group matched to comparison group(s) by chronological age; ^MA^, ASD group matched to comparison group(s) by mental age; ^O^, Statistic refers to *N* overall; ^*^, Autism, autistic disorder, or PDD groups were combined to create ASD group; ^**^, Information reflects only ASD group; ^
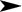
^, TD children, non-ASD children, lower-familial-likelihood siblings, and/or higher-familial-likelihood siblings without ASD were combined to create TD group. Measures: ADI-R, Autism Diagnostic Interview-Revised; ADOS, Autism Diagnostic Observation Schedule; BISCUIT, Baby and Infant Scale for Children with aUtIsm Traits; DBC-P, Developmental Behavior Checklist-Primary Carer Version; DPA, Developmental Play Assessment; ITSP, Infant/Toddler Sensory Profile; RBS-R, Repetitive Behavior Scale-Revised; RMRIS, Repetitive Movement and Restricted Interest Scales; RSMS, Repetitive and Stereotyped Movement Scales; SEQ, Sensory Experiences Questionnaire; SORF, Systematic Observation of Red Flags for Autism Spectrum Disorders in Young Children; SPA, Sensory Processing Assessment; SRS, Social Responsiveness Scale; SSP, Short Sensory Profile.

### RQ1: Comparisons of RRB across groups

Complete findings addressing RQ1 are presented in Supplementary Table 1 and are summarized below. Overall, far more studies examined RRB in autistic children in comparison to TD children (*n* = 24) than to children with DD (*n* = 13). The most frequently examined variables were RSM (*n* = 16), overall-RRB (*n* = 12), and sensory behaviors (*n* = 10), with comparisons of IS (*n* = 3) and SIB (*n* = 2) less common. Group differences in RSM, overall-RRB, and sensory behaviors were examined across the birth through age 3 developmental period, while group differences in IS and SIB were primarily focused on birth through age 2. In studies that address RQ1, SIB has only been measured indirectly; however, all other RRB categories have been measured using a combination of direct and indirect measures. Due to the low number of studies comparing SIB, these results are presented in Supplementary Table 1 but are not further interpreted in this section.

During the birth through age 3 developmental period, overall-RRB and RSM were significantly higher in autistic children than in TD children and children with DD. Comparisons of IS and sensory behaviors predominantly were mixed, indicating that autistic children either have similar (S1; S9; S31; S39; S41) or significantly higher (S3; S11; S18; S31; S33; S40; S41) IS or sensory behaviors relative to TD children and children with DD. One study (S1) found that children with ASD had significantly lower RSM (specifically object usage) than children with DD. However, no studies indicated that autistic children have less IS or sensory impairments than TD children or children with DD. These findings remained consistent across all ages and measurement approaches (i.e., direct or indirect).

### RQ2: Changes in RRB over time

Developmental changes in RRB by severity, frequency, and duration patterns over time were only reported in a subset of the longitudinal and repeated measures studies (*n* = 12). There were notable increases in severity, frequency, and duration for ASD groups. Increases in circumscribed interests, unusual preoccupations, compulsions, and rituals (IS), as well as hand and finger mannerisms and repetitive use of objects (RSM) and sensory behaviors were reported in four studies (S15; S36; S26; S40). Conversely, decreasing (S16; S19) or stable (S5; S18) levels of overall-RRB were reported in three studies, while two studies reported decreasing or stable trajectories in specific topographies [i.e., motor stereotypies (S20), SIB (S36), and sensory seeking behavior (S41)]. One study reported relatively stable RSM and increasing IS (S32). One study reported increases in overall duration of RRB over time (S17).

### RQ3: Relations between RRB and child outcomes

Findings addressing RQ3 are presented in Table 3. The RRB categories most frequently examined in relation to child outcomes were RSM (*n* = 13) and overall-RRB (*n* = 12), followed by IS (*n* = 5), sensory behaviors (*n* = 5), and SIB (*n* = 4). The child outcomes most frequently examined were cognitive ability (*n* = 19), social-communication/interaction (*n* = 18), and RRB severity (*n* = 14), followed by adaptive function (*n* = 10), ASD severity (*n* = 6), motor ability (*n* = 3), and self-regulation (*n* = 2). In studies that address RQ3, all RRB categories were measured using a mix of direct and indirect measures and outcome measures were both concurrent and distal. Due to the low number of studies examining motor ability and self-regulation, their relations with RRB categories are presented in Table 3 but are not further interpreted in this section.

**Table 3:** Relations between RRB and early childhood outcomes.

**RRB domain**	**Outcome**
**ASD severity**	
Overall-RRB	**25.7m:** ADOS RRB scores were +L correlated with ADOS total scores for both male and female participants (S19).
RSM	**19.8m:** RMRIS total scores (of CSBS) at age 2 were not correlated with age 3 ADOS total scores. Based on RMRIS of CSBS, repetitive movements with objects at age 2 were +M correlated with age 3 ADOS total scores. Repetitive movements of body, clutching objects, and sticky attention, excessive interest, dysregulation over object removal, and sensory interest were not correlated with age 3 ADOS RRB scores. RMRIS total scores (of home observations) at age 2 were not correlated with age 3 ADOS total scores. Based on RMRIS of home observations, repetitive movements with objects, repetitive movements of body, clutching objects, and sticky attention, excessive interest, dysregulation over object removal, and sensory interest were not correlated with age 3 ADOS RRB scores (S35). **26.2m:** Presence of preoccupations with abnormal intensity/focus, preoccupation with parts of objects, and stereotyped/repetitive motor mannerisms (based on review of ADI-R, ADOS, clinical judgment) at age 1–2 and 3–5 were not significant predictors of age 8–10 ADOS or CARS total scores (S36).
IS	**26.2m:** Presence of adherence to routines/rituals (based on review of ADI-R, ADOS, clinical judgment) at age 1–2 and 3–5 were not significant predictors of age 8–10 ADOS or CARS total scores (S36).
SIB	**26.2m:** Presence of SIB (based on review of ADI-R, ADOS, clinical judgment) at age 1–2 and 3–5 were not significant predictors of age 8–10 ADOS or CARS total scores (S36).
Sensory	**12.7m:** SEQ total, hyper-responsivity, hypo-responsivity, sensory-seeking, auditory modality, tactile modality, and visual modality scores at 12 months were not correlated with 24 month ADOS severity scores (S41). **14.0m:** Hyporeactivity (SPA and SEQ) at 14 months and changes in hyporeactivity from 14 to 23 months were not correlated with preschool-age ADOS total scores. Hyperreactivity (SPA and SEQ) at 14 months and changes in hyperreactivity (SPA and SEQ) from 14 to 23 months were not correlated with preschool-age ADOS total scores. Sensory seeking (SPA and SEQ) at 14 months and changes in sensory seeking (SPA and SEQ) from 14 to 23 months were not correlated with preschool-age ADOS total scores (S13). **26.2m:** Presence of sensory interests (based on review of ADI-R, ADOS, clinical judgment) at age 1–2 was not a significant predictor of age 8–10 ADOS or CARS total scores. Presence of sensory interests (based on review of ADI-R, ADOS, clinical judgment) at age 3–5 was positively associated with age 8–10 CARS (but not ADOS) total scores (S36). **33.7m:** SSP total sensory scores were not correlated with ADI-R total scores (S33).
**RRB severity**	
Overall-RRB	**25.5m:** RBS-R total scores were not correlated with ADOS-T RRB scores (S34). **26.4m:** ADOS RRB scores at age 2 were +S correlated with adolescent ADOS RRB scores but not SRS restricted interests and repetitive behavior scores (S5). **28.4m:** ADOS RRB scores at 12–30 months were positively associated with ADOS RRB scores at 31–56 months, controlling for MSEL NVIQ, diagnosis, age, and gender at 31–56 months (S18). **45.5m:** ADOS-G RRB scores at T1 (45.5 months) were +M correlated with frequency of observed RRB concurrently and at T3 (13 months later) but not at T2 (7 months later). ADI-R RRB scores at T1 were not correlated with observed RRB at any time point (S15).
RSM	**19.8m:** RMRIS total scores (of CSBS) at age 2 were not correlated with age 3 ADOS RRB scores. Based on RMRIS of CSBS, repetitive movements with objects at age 2 were +M correlated with age 3 ADOS RRB scores but excessive interest, repetitive movements of body, clutching objects, sticky attention, dysregulation over object removal, and sensory interest at age 2 were not correlated with age 3 ADOS RRB scores. RMRIS total scores (of home observations) at age 2 were +M correlated with age 3 ADOS RRB scores. Based on RMRIS of home observations, excessive interest at age 2 were +M correlated with age 3 ADOS RRB scores but repetitive movements with objects, repetitive movements of body, clutching objects, sticky attention, dysregulation over object removal, and sensory interest at age 2 were not correlated with age 3 ADOS RRB scores (S35). **21.3m:** RSMS composite and RSMS RRB with body cluster scores at age 2 were +M correlated with age 4 ADOS RRB score but RSMS RRB with objects cluster scores were not (S27). **21.3m:** Frequency and duration of observed RRB with objects and observed RRB with body at age 2 were not correlated with age 4 ADOS RRB scores, controlling for age (S38). **25.5m:** RBS-R stereotyped subscale scores were +S correlated with ADOS-T RRB scores, but RBS-R restricted subscale scores were not (S34). **26.2m:** Presence of preoccupations with abnormal intensity/focus, preoccupation with parts of objects, and stereotyped/repetitive motor mannerisms (based on review of ADI-R, ADOS, clinical judgment) at age 1–2 were not significant predictors of age 8–10 RBS-R total scores. Only preoccupation with abnormal intensity/focus at age 3–5 was positively associated with age 8–10 RBS-R total scores (S36).
	**40.7m:** RSM (RBS-R) was +S/+M correlated with ADOS and ADI-R RRB scores (S23). **47.1m:** Frequency of observed repetitive behaviors overall and frequency of observed object exploration overall were not correlated. Duration of observed repetitive behaviors overall and duration of observed object exploration overall were also not correlated (S17).
IS	**25.5m:** RBS-R ritualistic/sameness and compulsive subscale scores were not correlated with ADOS-T RRB scores (S34). **26.2m:** Presence of adherence to routines/rituals (based on review of ADI-R, ADOS, clinical judgment) at age 1–2 was not a significant predictor of age 8–10 RBS-R total scores. Presence of adherence to routines/rituals (based on review of ADI-R, ADOS, clinical judgment) at age 3–5 was positively associated with age 8–10 RBS-R total scores (S36). **40.7m:** IS (RBS-R) was +M correlated with ADI-R RRB scores but not ADOS RRB scores (S23).
SIB	**25.5m:** RBS-R SIB subscale scores were not correlated with ADOS-T RRB scores (S34). **26.2m:** Presence of SIB (based on review of ADI-R, ADOS, clinical judgment) at age 1–2 and 3–5 were not significant predictors of age 8–10 RBS-R total scores (S36). **40.7m:** SIB (RBS-R) was +S correlated with ADI-R RRB scores but not ADOS RRB scores (S23)
Sensory	**12.7m:** Most SEQ scores (total, hyper-responsivity, hypo-responsivity, sensory-seeking, auditory modality, tactile modality, and visual modality scores) at 12 months were +S/L correlated with 12- and 24-month RBS-R stereotyped, ritualistic/sameness, restricted, and SIB subscale scores (S41). **14.0m:** Hyporeactivity (SPA and SEQ) at 14 months was not correlated with preschool-age ADOS RRB scores. Changes in hyporeactivity (SPA but not SEQ) from 14 to 23 months was +S correlated with preschool-age ADOS RRB scores. Hyperreactivity (SPA) at 14 months and changes in hyperreactivity (SPA and SEQ) from 14 to 23 months were not correlated with preschool-age ADOS RRB scores. Hyperreactivity at 14 months (SEQ) was +M correlated with preschool-age ADOS RRB scores. Sensory seeking (SPA and SEQ) at 14 months and changes in sensory seeking (SPA and SEQ) from 14 to 23 months were not correlated with preschool-age ADOS RRB scores (S13). **26.2m:** Presence of sensory interests (based on review of ADI-R, ADOS, clinical judgment) at age 1–2 and 3–5 were not significant predictors of age 8–10 RBS-R total scores (S36). **33.7m:** SSP total sensory scores were +M correlated with ADOS RRB scores but not ADI-R RRB scores (S33).
**Social-communication/interaction**	
Overall-RRB	**12.6m:** RBS-R total inventory scores were not correlated with VABS-II socialization scores at 12 months but were –M correlated at 24 months (S40). **25.5m:** RBS-R total scores were not correlated with VABS-II socialization or ADOS-T social affect scores (S34). **25.7m:** ADOS RRB scores were +M correlated with ADOS social affect scores for male participants but not for female participants (S19). **26.4m:** ADOS RRB scores at age 2 were –S/–M correlated with adolescent VABS communication and socialization scores and +S correlated with adolescent ADOS social affect scores but not adolescent SRS social communication scores (S5). **31.0m:** At age 2, ADOS RRB scores were –M correlated with PLS-4 auditory comprehension scores but not PLS-4 expressive communication scores, controlling for chronological age. At age 3, ADOS RRB scores were –M correlated with both outcomes, controlling for chronological age. Decreases in ADOS RRB scores from age 2–3 were –M correlated with gains in both outcomes from age 2–3. Both outcomes were significant predictors of decreases in ADOS RRB scores even when controlling for chronological age, cognitive ability, and time between visits (S30). **31.6m:** ADOS RRB scores were –S correlated with ADOS social affect scores (S37). **37.1m:** ADI-R RRB scores at T1 (2–4 years) were –M/–L correlated with T1 VABS communication and socialization scores and MSEL receptive and expressive language scores. ADI-R RRB scores at T2 (13 months later) were –S/M correlated with all outcomes at T2 except MSEL expressive language (S16). **38.1m:** Lower-level RRB factor (ADI-R) was –S correlated with VABS communication scores. Higher-level (ADI-R) RRB factor (ADI-R) was not (S25). **45.5m:** PLS language scores at T1 (45.5 months) were –M correlated with frequencies of observed RRB concurrently, at T2 (7 months later), and at T3 (13 months later). ADOS-G social affect scores and ADI-R communication and reciprocal social interaction subdomain scores at T1 were not correlated with observed RRB at any time point (S15).
RSM	**12.6m:** RBS-R restricted and stereotyped subscale inventory scores were not correlated with VABS-II socialization scores at 12 months but both were –L correlated with VABS-II socialization scores at 24 months (S40). **19.8m:** RMRIS total scores (of CSBS) at age 2 were not correlated with age 3 ADOS social affect scores. Based on RMRIS of CSBS, repetitive movements with objects at age 2 were –M correlated with concurrent CSBS social (but not CSBS speech) composite scores and +M correlated with age 3 ADOS social affect scores. Excessive interests, repetitive movements of body, clutching, and sticky attention, dysregulation over object removal, and sensory interest at age 2 were not correlated with any outcome.
	RMRIS total scores (of home observations) at age 2 were not correlated with age 3 ADOS social affect scores. Based on RMRIS of home observations, excessive interests at age 2 was –S/M correlated with concurrent CSBS social and speech composite scores but not age 3 ADOS social affect scores. Repetitive movements with objects, repetitive movements of body, clutching, and sticky attention, dysregulation over object removal, and sensory interest at age 2 were not correlated with any outcome (S35). **21.3m:** RSMS RRB with objects cluster, RSMS RRB with body cluster, and RSMS composite scores at age 2 were –M/–L correlated with concurrent CSBS speech and social composite scores. Only RSMS RRB with objects cluster scores at age 2 were +S correlated with age 4 ADOS social affect scores. (S27). **21.3m:** Frequency and duration of observed RRB with objects at age 2 were –M correlated with concurrent CSBS social composite scores but not age 4 ADOS social affect scores, controlling for age. Frequency and duration of observed RRB with body at age 2 were not correlated with either outcomes, controlling for age (S38). **25.5m:** RBS-R stereotyped and restricted subscale scores were –S/M correlated with VABS-II socialization scores. RBS-R stereotyped subscale scores were +S correlated with ADOS-T social affect scores but RBS-R restricted subscale scores were not (S34). **28.9m:** Changes in RSM scores (ADI-R) from age 2–9 were not correlated with age 2 ADOS social affect scores in a model controlling for age, diagnosis, sex, race, maternal education, site, and age 2 MSEL NVDQ scores (S32). **33.6m:** Restricted object use (DPA) at ~3 years was –M correlated with response to joint attention and coordinated attention to object and person concurrently and 6 months later (S6). **40.7m:** RSM (RBS-R) was +S correlated with ADI-R social and communication scores (S23).
IS	**12.6m:** At 12 and 24 months, RBS-R ritualistic/sameness and compulsive subscale inventory scores were not correlated with VABS-II socialization scores (S40). **25.5m:** RBS-R ritualistic/sameness and compulsive subscale scores were –S correlated with VABS-II socialization scores but not ADOS-T social scores (S34). **28.9m:** Changes in IS scores (ADI-R) from age 2–9 were negatively associated with age 2 ADOS social affect scores in a model controlling for age, diagnosis, sex, race, maternal education, site, and age 2 MSEL NVDQ scores (S32). **40.7m:** IS (RBS-R) was +S correlated with ADI-R social and communication scores (S23).
SIB	**12.6m:** At 12 and 24 months, RBS-R SIB subscale inventory scores were not correlated with VABS-II socialization scores (S40). **25.5m:** RBS-R SIB subscale scores were –M correlated with VABS-II socialization scores but not ADOS-T social affect scores (S34). **40.7m:** SIB (RBS-R) was +S correlated with ADI-R social and communication scores (S23).
Sensory	**12.7m:** SEQ total, hyper-responsivity, hypo-responsivity, sensory-seeking, auditory modality, tactile modality, and visual modality scores at 12 months were –M/–L correlated with 24 month VABS-II socialization scores. SEQ total, hypo-responsivity, sensory-seeking, auditory modality, and visual modality (but not hyper-responsivity and tactile modality) scores at 12 months were –M/–L correlated with 24 month VABS-II communication scores (S41). **14.0m:** Hyporeactivity (SPA) at 14 months and changes in hyporeactivity (SPA) from 14 to 23 months was +S/M correlated with preschool-age ADOS social affect scores. Hyporeactivity (SEQ) at 14 months and changes in hyporeactivity (SEQ) from 14 to 23 months were not correlated. Hyperreactivity (SPA and SEQ) at 14 months and changes in hyperreactivity (SPA and SEQ) from 14 to 23 months were not correlated with preschool-age ADOS social affect scores. Sensory seeking (SPA and SEQ) at 14 months and changes in sensory seeking (SPA and SEQ) from 14 to 23 months were not correlated with preschool-age ADOS social affect scores (S13). **33.7m:** SSP total sensory scores were not correlated with ADI-R communication and social scores or ADOS social affect scores (S33).
**Cognitive ability**	
Overall-RRB	**12.6m:** At 12 and 24 months, RBS-R total inventory scores were not correlated with MSEL ELC or NVDQ scores (S40). **25.5m:** RBS-R total scores were not correlated with MSEL ELC or NVDQ scores (S34). **25.7m:** ADOS RRB scores were –M/L correlated with MSEL NVDQ, VDQ, and overall scores for male participants but not for female participants (S19). **26.4m:** ADOS RRB scores at age 2 were –M correlated with adolescent WISC-IV/WAIS-IV IQ scores. ADOS RRB scores at age 2 were also negatively associated with adolescent WISC-IV/WAIS-IV IQ scores in a model controlling for age, sex, IQ, and ADOS social affect scores at age 2 (S5). **28.4m:** MSEL NVDQ scores were negatively associated with ADOS RRB scores for children with PDD-NOS ≥25 months but not for children with PDD-NOS < 25 months. MSEL NVDQ scores were not associated with ADOS RRB scores for children with autism at any age (S18).
	**31.0m:** At age 2 and 3, ADOS RRB scores were –M correlated with MSEL visual reception scores, controlling for chronological age. Changes in ADOS RRB scores from age 2–3 were not correlated with changes in this outcome from age 2–3 (S30). **31.6m:** ADOS RRB scores were –S correlated with MSEL NVDQ but not MSEL VDQ scores (S37). **36.8m:** Developmental age (PEP-R) did not predict ADI-R RRB scores, higher-level RRB scores (ADI-R), lower-level RRB scores (ADI-R), or DBC-P RRB scores, controlling for VABS ABC scores (S24). **37.3m:** Higher frequencies of observed RRB were –M/L correlated with non-verbal but not verbal abilities (MSEL) in both male and female participants (S14). **38.1m:** Higher-level RRB factor (ADI-R) was +S correlated with developmental age (PEP-R) while lower-level RRB factor (ADI-R) was –M correlated (S25). **45.5m:** MSEL NVIQ scores at T1 (45.5 months) were –M correlated with frequencies of observed RRB concurrently, at T2 (7 months later), and at T3 (13 months later) (S15).
RSM	**12.6m:** At 12 months, RBS-R restricted subscale inventory scores were –M correlated with MSEL ELC and NVDQ scores but RBS-R stereotyped subscale inventory scores were not. At 24 months, neither RBS-R subscale inventory scores were correlated with MSEL ELC or NVDQ scores (S40). **19.8m:** RMRIS total scores (of CSBS) at age 2 were –M correlated with age 3 MSEL NVDQ (but not VDQ) scores. Based on RMRIS of CBSS, repetitive movements with objects at age 2 were –M/L correlated with concurrent CSBS symbolic scores and age 3 MSEL NVDQ but not VDQ scores. Repetitive movements of body, excessive interest, and sticky attention, clutching objects, dysregulation over removal of object, and sensory interest at age 2 were not correlated with any outcome. RMRIS total scores (of home observations) at age 2 were not correlated with age 3 MSEL NVDQ or VDQ scores. Based on RMRIS of home observations, clutching objects at age 2 was –M correlated with age 3 MSEL VDQ but not NVDQ or concurrent CSBS symbolic scores. Repetitive movements of body, excessive interest, and sticky attention, repetitive movements with objects, dysregulation over removal of object, and sensory interest at age 2 were not correlated with any outcome (S35). **21.3m:** RSMS composite scores at age 2 were –M correlated with concurrent CSBS symbolic scores but RSMS RRB with objects and RSMS RRB with body cluster scores at age 2 were not. RSMS composite scores at age 2 were –M correlated with age 4 MSEL NVDQ and VDQ scores. RSMS RRB with objects cluster scores at age 2 were –M correlated with both outcomes. RSMS RRB with body cluster scores at age 2 were not correlated with either outcome (S27). **21.3m:** Frequency and duration of observed RRB with objects at age 2 were –M correlated with concurrent CSBS symbolic scores and age 4 MSEL NVDQ and VDQ scores, controlling for age. Frequency and duration of observed RRB with body at age 2 were not correlated with any outcomes, controlling for age (S38). **25.5m:** RBS-R stereotyped subscale scores were –S correlated with MSEL ELC and NVDQ scores but RBS-R restricted subscale scores were not (S34).
	**26.2m:** Presence of preoccupation with abnormal intensity/focus, preoccupation with parts of objects, and stereotyped/repetitive motor mannerisms (based on review of ADI-R, ADOS, clinical judgment) at age 1–2 were not significant predictors of age 8–10 DAS-II verbal or non-verbal reasoning. Only presence of preoccupation with parts of objects at age 3–5 was negatively associated with age 8–10 verbal (but not non-verbal) reasoning (S36). **28.9m:** Changes in RSM scores (ADI-R) from age 2–9 were negatively associated with age 2 MSEL NVDQ scores in a model controlling for age, diagnosis, sex, race, maternal education, site, and age 2 ADOS social affect scores (S32). **37.3m:** Higher frequencies of observed object RRB were –M correlated with non-verbal and verbal abilities (MSEL) in male but not in female participants (S14). **40.7m:** RSM (RBS-R) was not correlated with M-P-R developmental index (S23).
IS	**12.6m:** At 12 and 24 months, RBS-R ritualistic/sameness and compulsive subscale inventory scores were not correlated with MSEL ELC or NVDQ scores (S40). **25.5m:** RBS-R ritualistic/sameness and compulsive subscale scores were not correlated with MSEL ELC or NVDQ scores (S34). **26.2m:** Presence of adherence to routines/rituals (based on review of ADI-R, ADOS, clinical judgment) at age 1–2 and 3–5 were not significant predictors of age 8–10 DAS-II verbal or non-verbal reasoning (S36). **28.9m:** Changes in IS scores (ADI-R) from age 2–9 were not associated with age 2 MSEL NVDQ scores in a model controlling for age, diagnosis, sex, race, maternal education, site, and age 2 ADOS social affect scores (S32). **40.7m:** IS (RBS-R) was not correlated with M-P-R developmental index (S23).
SIB	**12.6m:** At 12 and 24 months, RBS-R SIB subscale inventory scores were not correlated with MSEL ELC or NVDQ scores (S40). **25.5m:** RBS-R SIB subscale scores were not correlated with MSEL ELC or NVDQ scores (S34).
	**26.2m:** Presence of SIB (based on review of ADI-R, ADOS, clinical judgment) at age 1–2 and 3–5 were not significant predictors of age 8–10 DAS-II verbal or non-verbal reasoning (S36). **40.7m:** SIB (RBS-R) was not correlated with M-P-R developmental index (S23).
Sensory	**12.7m:** SEQ total, hyper-responsivity, hypo-responsivity, sensory-seeking, auditory modality, tactile modality, and visual modality scores at 12 months were not correlated with MSEL ELC at 24 months (S41). **26.2m:** Presence of sensory interests (based on review of ADI-R, ADOS, clinical judgment) at age 1–2 was not a significant predictor of age 8–10 DAS-II verbal or non-verbal reasoning. Presence of sensory interests (based on review of ADI-R, ADOS, clinical judgment) at age 3–5 was negatively associated with age 8–10 non-verbal (but not verbal) reasoning (S36). **33.7m:** SSP total sensory scores were not correlated with mental age (MSEL) (S33). **37.3m:** Frequencies of observed visual RRB were not correlated with non-verbal or verbal abilities (MSEL) for male or female participants (S14).
**Adaptive function**	
Overall-RRB	**12.6m:** RBS-R total inventory scores were not correlated with VABS-II ABC scores at 12 months but were –M correlated at 24 months (S40). **25.5m:** RBS-R total scores were –M correlated with VABS-II ABC scores (S34). **26.4m:** ADOS RRB scores at age 2 were –M correlated with adolescent VABS daily living skills scores. ADOS RRB scores at age 2 were not associated with adolescent VABS ABC scores in a model controlling for age, sex, WISC-IV/WAIS-IV IQ, and ADOS social affect scores at age 2 (S5). **31.6m:** ADOS RRB scores were –S correlated with VABS-II ABC scores (S37). **36.8m:** VABS ABC scores was negatively associated with DBC-P RRB scores but did not predict ADI-R RRB scores, higher-level RRB scores (ADI-R), or lower-level RRB scores (ADI-R), controlling for severity of developmental delay and either chronological or developmental age (PEP-R) (S24). **38.1m:** Lower-level RRB factor (ADI-R) was –S correlated with VABS ABC scores. Higher-level RRB factor (ADI-R) was not (S25).
RSM	**12.6m:** RBS-R restricted and stereotyped subscale inventory scores at 12 months were not correlated with VABS-II ABC scores but were –M correlated at 24 months (S40). **21.3m:** Frequency and duration of observed RRB with objects and observed RRB with body at age 2 were not correlated with age 4 VABS ABC scores, controlling for age (S38). **25.5m:** RBS-R stereotyped subscale scores were –M correlated with VABS-II ABC scores but RBS-R restricted subscale scores were not (S34). **26.2m:** Presence of preoccupation with abnormal intensity/focus, preoccupation with parts of objects, and stereotyped/repetitive motor mannerisms (based on review of ADI-R, ADOS, clinical judgment) at age 1–2 and 3–5 were not significant predictors of age 8–10 VABS-II ABC scores (S36). **40.7m:** RSM (RBS-R) was –S correlated with VABS-II ABC scores (S23).
IS	**12.6m:** RBS-R ritualistic/sameness and compulsive subscale inventory scores were not correlated with VABS-II ABC scores at 12 months but RBS-R compulsive subscale inventory scores were –M correlated at 24 months (S40). **25.5m:** RBS-R ritualistic/sameness and compulsive subscale scores were not correlated with VABS-II ABC scores (S34).
	**26.2m:** Presence of adherence to routines/rituals (based on review of ADI-R, ADOS, clinical judgment) at age 1–2 and 3–5 were not significant predictors of age 8–10 VABS-II ABC scores (S36). **40.7m:** IS (RBS-R) was –S correlated with VABS-II ABC scores (S23).
SIB	**12.6m:** At 12 and 24 months, RBS-R SIB subscale inventory scores were not correlated with VABS-II ABC scores (S40). **25.5m:** RBS-R SIB subscale scores were –S correlated with VABS-II ABC scores (S34). **26.2m:** Presence of SIB (based on review of ADI-R, ADOS, clinical judgment) at age 1–2 and 3–5 were not significant predictors of age 8–10 VABS-II ABC scores (S36). **40.7m:** SIB (RBS-R) was –S correlated with VABS-II ABC scores (S23).
Sensory	**12.7m:** SEQ total, hyper-responsivity, hypo-responsivity, sensory-seeking, auditory modality, tactile modality, and visual modality scores at 12 months were not correlated with VABS-II ABC scores at 24 months (S41). **26.2m:** Presence of sensory interests (based on review of ADI-R, ADOS, clinical judgment) at age 1–2 and 3–5 were not significant predictors of age 8–10 VABS-II ABC scores (S36). **33.7m:** SSP total sensory scores explained significant additional variance in VABS-II ABC scores (4%) when added to model with MSEL ELC (S33).
**Motor**
**Overall-RRB**	
RSM	**12.7m:** RSMS RRB with body scores were –M correlated with MSEL gross motor scores. RSMS RRB with object scores were not correlated (S10). **33.6m:** Restricted object use (DPA) at ~3 years was –M correlated with motor imitation concurrently and 6 months later (S6).
**IS**	
**SIB**	
Sensory	**12.7m:** SEQ total, hyper-responsivity, hypo-responsivity, sensory-seeking, auditory modality, tactile modality, and visual modality scores at 12 months were not correlated with VABS-II motor scores at 24 months (S41).
**Self-Regulation**	
Overall-RRB	**31.6m:** ADOS RRB scores were not correlated with CBCL dysregulated profile scores or ADOS aggressive-disruptive scores (S37).
RSM	**40.7m:** RSM (RBS-R) was +M/L correlated with CBCL internalizing, externalizing, and total problems raw scores (S23).
IS	**40.7m:** IS (RBS-R) was +L correlated with CBCL internalizing, externalizing, total problems, and pervasive developmental problems raw scores (S23).
SIB	**40.7m:** SIB (RBS-R) was +M correlated with CBCL internalizing, externalizing, total problems, and pervasive developmental problems raw scores (S23).
Sensory	

Ages refer to mean age (months) of ASD sample (at intake/Time 1) within each study. Interpretation of correlations follow Cohen (1988; small = < 0.30, medium = 0.30–0.49, large = < 0.50); +S, significant-positive-small correlation; +M, significant-positive-medium correlation; +L, significant-positive-large correlation; –S, significant-negative-small correlation; –M, significant-negative-medium correlation; –L, significant-negative-large correlation. Measures: ADI-R, Autism Diagnostic Interview-Revised; ADOS, Autism Diagnostic Observation Schedule; CARS, Childhood Autism Rating Scale; CBCL, Child Behavior Checklist; CSBS, Communication and Symbolic Behavior Scales; DAS-II, Differential Ability Scales; DBC-P, Developmental Behavior Checklist-Primary Carer Version; DPA, Developmental Play Assessment; M-P-R, Merrill-Palmer-Revised Scales of Development; MSEL, Mullen Scales of Early Learning; PEP-R, Psychoeducational Profile-Revised; PLS-4, Preschool Language Scale Fourth Edition; RBS-R, Repetitive Behavior Scale-Revised; SEQ, Sensory Experiences Questionnaire; SSP, Short Sensory Profile; VABS, Vineland Adaptive Behavior Scales; WAIS-IV, Wechsler Adult Intelligence Scale; WISC-IV, Wechsler Intelligence Scale for Children.

#### RRB categories in relation to ASD severity

Three studies did offer some evidence that, in general, more severe RRB was associated with higher ASD severity: S19 found that ADOS RRB scores were positively correlated with ADOS total scores for both male and female participants; S35 found that repetitive movements with objects (based on the RMRIS of CSBS) at age 2 was positively correlated with age 3 ADOS total scores; and S36 found that presence of sensory interests at age 3–5 was positively correlated with CARS total scores from ages 8 through 10.

#### RRB categories in relation to RRB severity

Studies revealed mixed findings, indicating either no relations or more commonly positive relations (i.e., in general greater frequency or severity of RRB was associated with more severe concurrent or distal RRB). No studies indicated negative relations wherein greater RRB was associated with less frequent or severe concurrent or distal RRB.

#### RRB categories in relation to social-communication/interaction

Study findings were mixed, indicating either no or negative relations (i.e., in general greater frequency or severity of RRB was associated with lower social-communication/interaction outcomes).

#### RRB categories to in relation cognitive ability

The studies revealed mixed findings regarding the relations of overall-RRB to cognitive ability and RSM to cognitive ability, indicating either no or less commonly negative relations (i.e., higher overall-RRB/RSM were associated with lower cognitive ability). Restricted and repetitive behaviors by age 3 were significantly negatively correlated with non-verbal cognitive skills (S30) and also significantly negatively associated with stereotyped behavior in particular (S34). Cognitive ability was generally shown to be unrelated to IS, SIB, and sensory behaviors. Only one study (S25) found a positive relation between higher-order RRB (as measured by the ADI-R) and cognitive ability.

#### RRB categories and adaptive function

Study findings were mixed, indicating either no or negative relations between RRB and adaptive function. In general, greater frequency or severity of RRB was associated with lower adaptive function.

Findings of relations between each of the five RRB categories and child outcomes remained consistent whether direct or indirect measures of RRB were used and with participant samples spanning the birth through age 3 developmental period. With the exception of relations between RRB categories and ASD severity, which were predominantly examined distally, relations between RRB categories and all other child outcomes were examined both concurrently and distally.

## Discussion

We conducted a systematic review of studies examining RRB in autistic children from birth through age 3 to clarify what is currently known of the distinctiveness of RRB in young autistic children, the developmental trajectory of RRB in early childhood, and the relations between RRB and child outcomes. We identified 41 studies meeting inclusion criteria for review, including 12 studies containing longitudinal data. We found that existing studies suggest that RRB frequency and severity during the period of birth through age 3 are significantly higher in autistic children in comparison to TD children and children with DD, though differences for the latter comparison were less consistent. While the studies we reviewed relied on a range of measurement approaches, there was in general agreement across indirect (e.g., parent report) and direct observational measures. Of note, there was converging evidence from multiple studies that increased RRB associated with ASD is evident during early toddlerhood and prior to age 2. While there were no studies identified in our sample examining RRB prior to 12 months of age, one paper published since our search closed reported elevated RSM in children later diagnosed with ASD at age 9 months (Miller et al., 2021). This contributes additional evidence suggesting that elevated RRB may be an early behavioral marker of ASD.

We further found that most topographies of RRB (i.e., RSM, IS, SIB, sensory) were largely unrelated to autism severity during the first years of life. This suggests that many varieties of repetitive behavior may be seen in young children who have or are presymptomatic/prodromal for ASD irrespective of degree of clinical severity. This is in contrast to findings from studies of older children, where modest relations between multiple forms of RRB and ASD severity have been reported (e.g. Uljarević et al., 2022). Interestingly, we did see some evidence that RRB was negatively associated with language skills (e.g., S15; S16; S30), though relatively few studies examined these relations. Similar to autism severity, we found that IS, SIB, and sensory behaviors were largely unrelated to cognitive ability. This pattern was observed across multiple studies and stands in contrast to what has been reported in studies of older children and adults (e.g., Gabriels et al., 2005; Esbensen et al., 2009; Gotham et al., 2013). In those studies, RRB is reported to be negatively associated with cognitive function (Duerden et al., 2012). However, consistent with findings from older children (e.g. Lam et al., 2008), six studies reported associations between RSM and cognitive ability (S14; S27; S32; S34; S35, S38), though three others reported no such association (S23; S36; S40). Findings related to adaptive function were particularly mixed with some studies reporting no association and others reporting a small to moderate negative association, though no study reported a positive association between adaptive function and RRB.

It is notable that numerous studies included in our review consistently reported no association between RRB and either cognitive and adaptive function. We posit that the apparent inconsistency concerning relations of RRB to symptom severity, cognitive, and adaptive function likely reflects developmental effects whereby the range of RRB displayed by young children may be much broader and less reflective of other clinical features (Esbensen et al., 2009). Such associations likely emerge later as symptoms consolidate and as normative aspects of RRB lessen over early childhood. During toddlerhood, RRB associated with autism may occur at higher rates than in other children but may nonetheless serve similar adaptive functions. We did identify some evidence to this effect in the reviewed literature, wherein relations with RRB either emerged at later ages within a longitudinal sample (e.g., S36, S40) or were present in older toddlers/preschoolers but not in younger toddlers (e.g., S18).

There were several methodological factors that may also contribute to mixed findings. First, the studies we reviewed varied in sample characteristics (e.g., age, sex ratio, prospective vs. higher-familial-likelihood sample) and design (e.g., prospective, retrospective, longitudinal, cross sectional). Relatedly, a variety of direct and indirect RRB measures were used between and within studies. For example, S34 found that overall-RRB, as measured by the RBS-R, was negatively correlated with VABS ABC scores, while S24 found that overall-RRB, as measured by the ADI-R, was not related to VABS ABC scores. S35 looked at relations between RSM (based on RSMS of CSBS video samples vs. home video samples) and MSEL NVDQ and VDQ. They found that RSM, as measured by the RSMS on CSBS video samples, was negatively correlated with MSEL NVDQ, while RSM, as measured by the RSMS on home video samples, was not correlated with either MSEL outcomes. This suggests that both measurement and context may be important sources of variability. As such, it may be advisable to operationalize RRB constructs by combining measurement approaches.

A wide range of measures were utilized across the studies we reviewed, and it is clear there is no single standard measure of RRB. Variability in measurement approaches somewhat hinders attempts to synthesize the RRB literature around specific subtypes given that different research groups define and measure constructs differently. This was particularly true for RSM, IS, and sensory behavior subcategories. For example, studies focused on RSM examined topographies spanning items such as “repetitive movements with objects,” “repetitive movements of body,” “excessive interest,” “clutching objects,” “sticky attention,” “presence of preoccupations with abnormal intensity/focus,” “preoccupation with parts of objects,” “unusual posturing,” “repetitive leg and arm movements,” “twiddling/waving-,” “banging/tapping-,” “mouthing-,” “staring at/fixating-,” and “rubbing objects.” While these may all represent the construct of RSM, they are discrete topographies that most likely vary as a function of age and developmental level. Given this diversity of item-level topographies, coupled with differences in their definitions and measurements across research teams, nuanced findings regarding how RSM, IS, and sensory behaviors compare across ASD, TD, and DD groups and their relations to child outcomes are not surprising.

While existing findings suggest that RRB may differentiate autistic children from TD children by late infancy at the group level, the magnitude of such differences appears relatively modest. Indeed, most of the studies we reviewed, and in particular the longitudinal studies, suggest differences related to RRB become increasingly evident over toddlerhood. For example, S40 found that differences in RSM, IS, and SIB between autistic children and TD children became more pronounced at 24 relative to 12 months of age. They also found that RBS-R total inventory scores and subscales comprising the RSM and IS categories were associated with VABS ABC- and socialization scores at 24 months of age but that this association was not evident at 12 months of age. S36 found that presence of sensory interests at age 3–5 years, but not at age 1–2 years, were positively associated with CARS total scores and negatively associated with non-verbal reasoning at age 8–10 years. Likewise, adherence to routines/rituals at age 3–5 years, but not at age 1–2 years, were positively associated with RBS-R total scores at age 8–10 years. In summary, it is plausible that RRB in very early childhood inconsistently distinguish autistic children and are not yet predictive of child outcomes. Although there appears to be rationale for including RRB items in conjunction with others for the purpose of early screening, it would appear based on the literature to date that RRB alone are not sufficiently indicative of later outcomes in infants as currently measured. One issue for further study is whether alternative measurement approaches to RRB might offer greater sensitivity and predictive validity.

### Limitations

There are several limitations that merit consideration. First, this was not an exhaustive review of all studies that included data on RRB in autistic children from birth through age 3. Rather, we limited our sample to studies that provided data addressing our research questions. Therefore, the comparison groups and measurement strategies utilized varied across studies. Second, our review of research on some categories of RRB were limited to only a handful of published reports and as such, should be considered in aggregate with caution. For example, we did not interpret findings related to SIB given limited evidence. The handful of existing studies that examined SIB found that these behaviors do not differentiate those with ASD or DD in infancy; however, most measurement tools are not specific to early childhood. For example, self-directed motor behavior or proto-SIB (e.g., hitting head with hand in a manner unlikely to result in injury) may not rise to the level of a “problem” as measured by common instruments (e.g., ADI-R, RBS-R). Relatedly, there are potential sources of differences in findings related to control group type that we were unable to detect. Specifically, several studies utilized mental age matching to compose their comparison groups. Given the limited number of these studies and the heterogeneity among them, we were unable to conclude whether this factor was related to the strength and/or direction of study findings. Finally, there are aspects of our review criteria that may introduce bias. These include (1) restricting articles under consideration to English only, which over-represents predominantly White, Western populations and (2) searching databases of published articles, which introduces the potential for publication bias related to positive findings. Such sources of bias are not unique to this review but nonetheless warrant consideration.

### Conclusion and future directions

To better understand the utility of early RRB in informing early identification and intervention efforts, we synthesized the available literature on RRB in autistic children ages birth through three. We found that the frequency and severity of early RRB are significantly higher in autistic children in comparison to TD children and children with DD. Furthermore, there are marked differences in the developmental trajectory of RRB in autistic children in comparison to TD children during the period of birth through age 3. These findings suggest that both absolute level of current RRB as well as change in RRB over time may be useful in the early screening for ASD. Early RRB, however, may be less informative for predicting functional outcomes. We found no consistent relations between RRB categories and ASD severity and mixed relations between RRB categories and social communication/interactions, cognitive ability, and adaptive function, with several studies suggesting that these relations likely become stronger as children reach preschool age and beyond. Altogether, these findings suggest that RRB present in children birth through age 3 are not, by themselves, indicative of later functional outcomes, but may be informative in identifying autism regardless of level of ASD severity.

It is plausible that discrepancies in definitions and measures of RRB used across the literature may contribute to obscuring relations between this behavioral domain and other functional outcomes. Given the heterogeneity of behaviors included under the RRB domain in addition to the number of RRB definitions and measures used by research groups, it may be beneficial for researchers and practitioners to carefully consider measurement approaches used to capture early RRB, particularly as it relates to the many different behaviors that comprise the domain. To date, more research has examined RSM topographies than any other categories of RRB from birth to three, though the literature suggests other topographies may be important targets during this period of development. Finally, continuing to expand on the early RRB literature is important to attempt to address the variability of the results across studies. For instance, more precision around both measurement and aggregation of study samples is needed to interrogate contradictory findings in the future. Additional research is needed to further solidify our understanding of how atypical sensory behaviors, IS, and SIB develop during the first years of life in autistic children and the extent to which these may or may not impact quality of life.

## Data availability statement

The original contributions presented in the study are included in the article and Supplementary material. Further inquiries can be directed to the corresponding author.

## Author contributions

PC: conceptualization, data curation, investigation, methodology, validation, and writing—original draft preparation (lead). AD: conceptualization, data curation, investigation, methodology, validation, and writing—original draft preparation. JW: conceptualization, funding acquisition, methodology, supervision, and writing—review and editing. All authors contributed to the article and approved the submitted version.

## Funding

This work was supported by the National Institutes of Health under award R01MH116961.

## Conflict of interest

The authors declare that the research was conducted in the absence of any commercial or financial relationships that could be construed as a potential conflict of interest.

## Publisher's note

All claims expressed in this article are solely those of the authors and do not necessarily represent those of their affiliated organizations, or those of the publisher, the editors and the reviewers. Any product that may be evaluated in this article, or claim that may be made by its manufacturer, is not guaranteed or endorsed by the publisher.

## Author disclaimer

The content is solely the responsibility of the authors and does not necessarily represent the official views of the NIH.

## Footnotes

^1^We use identity-first language (e.g., autistic children) as opposed to person-first language (e.g., children with autism) in accordance with the American Psychological Association's (2021) inclusive language guidelines, which recommend usage of the language that an identity group prefers. Autistic individuals who have been able to have indicated a preference for identity-first language (e.g., Brown, 2011; Botha et al., 2021).
