# Supplementary material for: Restricted and repetitive behavior in children with autism during the first three years of life: A systematic review
Source: Front Psychol. 2022 Oct 31;13:986876. doi: 10.3389/fpsyg.2022.986876 (PMC9661964; doi:10.3389/fpsyg.2022.986876)
Supplement: Supplementary file 1 [file Table_1.DOCX]

Supplemental Table 1

*RRB in Children with ASD to Typically Developing Children and Children with Developmental Delays Birth Through Age 3*

| **RRB Type** | **ASD vs. TD** |
| --- | --- |
| **Overall-RRB** | **12.6m:** At 12 and 24 months, HL-ASD group had significantly higher RBS-R total inventory scores than HL-Neg and LL-Neg groups.  When assessed at 24 months, HL-ASD group also had significantly higher ADOS RRB scores than HL-Neg and LL-Neg groups (S40).  **19.1m:** ASD group demonstrated increased (worsening) ADOS RRB scores from T1 (15 - 24 months) to T2 (12 months later) whereas Non-ASD group demonstrated stability. The worsening of ADOS RRB scores for ASD group was accounted for by T1 MSEL NVQ (S12).  **20.7m:** ASD group had significantly higher ADOS-T RRB and SORF RRB scores (S9).  **21.0m:** At 20 and 42 months, ASD group had significantly higher ADI-R RRB scores (S7).  **21.0m:** ASD group had significantly higher SORF RRB scores (S39).  **~24m:** ASD group had a significantly higher mean number of endorsed ADI-R RRB items (S31).  **26.6m:** ASD group had significantly higher BISCUIT RRB scores (S22).  **28.4m:** ASD group had significantly higher ADOS RRB scores and prevalence of ADOS RRB items at <18, 19-24, 25-30, 31-36, 37-42, and 43-56 months, controlling for age, MSEL NVIQ, and gender. Severity of ADOS RRB scores were stable over time for ASD group but became less severe over time for TD group (S18).  **33.7m:** ASD group had significantly higher ADI-R and ADOS-G RRB scores (S33).  **37.7m:** HL-ASD group had significantly higher ADOS-2 RRB scores than HL-Neg and TD groups but similar RBS-R total scores (S28).  **45.5m:** ASD group had significantly higher frequencies of observed RRB at T1 (45.5 months), T2 (7 months later), and T3 (13 months later) (S15). |
| **RSM** | **9-12m:** ASD group had significantly higher rates of observed unusual posturing but similar rates of observed object play ratings, repetitive leg and arm movements, twiddling/waving-, banging/tapping-, mouthing-, staring at/fixating-, and rubbing objects (S1).  **12.0m:** ASD group displayed similar frequencies/durations of all observed typical object exploration behaviors: shaking/waving-, banging/tapping-, mouthing-, throwing/pushing object.  ASD group displayed significantly higher frequencies/durations of observed atypical object exploration behaviors: spinning objects, rotating objects, and unusual visual exploration of objects but a similar frequency of rolling objects (S29).  **12.1m:** At 12 months, ASD group had similar frequencies of observed finger extension, hands to ears, arm shake, arm wave, arm shake with object, arm wave with noisy object, arm bang on surface, and arm bang of objects together to HL-Neg group. At 18 months, ASD group had significantly higher frequencies of observed arm wave than HL-Neg group but nothing else.  At 12 months, ASD group had significantly higher frequencies of observed arm wave than LL-Neg group but similar frequencies of observed finger extension, hands to ears, arm shake, arm shake with object, arm wave with noisy object, arm bang on surface, and arm bang of objects together. At 18 months, ASD group had significantly higher frequencies of observed arm wave and hands to ears than LL-Neg groups but nothing else (S20).  **12.6m:** At 12 months, HL-ASD group had significantly higher RBS-R stereotyped and restricted subscale inventory scores than HL-Neg group. At 24 months, HL-ASD group had significantly higher RBS-R stereotyped subscale inventory scores but similar RBS-R restricted subscale inventory scores.  At 12 and 24 months, HL-ASD had significantly higher RBS-R stereotyped and restricted subscale inventory scores than LL-Neg group (S40).  **12.7m:** HL-ASD group had significantly higher RSMS composite and RSMS RRB w/ body cluster scores than HL-Neg but similar RSMS RRB w/ object cluster scores.  HL-ASD group had significantly higher RSMS composite, RSMS RRB w/ body, and RSMS RRB w/ object cluster scores than LL-Neg group.  When assessed at 24 months, HL-ASD group also had significantly higher ADOS RRB scores than HL-Neg and LL-Neg groups (S10).  **15.0m:** HL-ASD group had similar rates of RSMS RRB w/ body and RSMS RRB w/ objects to HL-Neg group. Both groups displayed significantly higher rates of RRB w/ objects than RRB w/ body.  HL-ASD group had a higher inventory of RSMS RRB w/ body and a lower inventory of RSMS RRB w/ objects than HL-Neg group (S8).  **20.7m:** ASD group had significantly higher scores on repetitive speech/intonation, repetitive use of objects, repetitive body movements, and excessive interest in particular objects, actions, or activities but similar scores on clutches particular objects, sticky attention to objects, and fixation on parts of objects (SORF) (S9).  **21.0m:** ASD group had significantly higher scores for repetitive movements/posturing of body, arms, hands, or fingers, repetitive movements with objects, and lack of playing with a variety of toys conventionally but similar scores for excessive interest in particular toys (SORF) (S39).  **21.3m:** ASD group had significantly higher frequency of observed RRB w/ objects overall. Specifically, ASD group demonstrated higher frequencies of rocking/flipping-, swiping-, rolling-, moving/placing-, clutching-, and spinning objects with small-medium effect sizes, but TD group demonstrated higher frequencies of banging/tapping-, shaking-, rubbing/squeezing-, lining up/stacking-, and collecting objects with small effect sizes.  ASD group had significantly higher frequency of observed RRB w/ body overall. Specifically, ASD group demonstrated higher frequencies of stiffening their fingers and patting-, rocking-, and rubbing their bodies with small-medium effect sizes, but TD group demonstrated higher frequency of banging their bodies against surface with a medium effect size.  ASD group had similarly low frequencies of observed sensory behaviors overall. Specifically, ASD group demonstrated higher frequencies of licking, feeling/touching, sucking fingers, and fixating with small effect sizes but demonstrated similar frequencies of sniffing/smelling and covering ears (S4).  **21.3m:** ASD group had significantly higher RSMS composite scores. Specifically, ASD group had significantly higher RSMS cluster scores, rates and inventories of RRB w/ objects; ASD group demonstrated more swiping-, rubbing/squeezing-, rolling/knocking-, rocking/flipping-, spinning/wobbling-, lining up/stacking-, moving/placing-, and clutching objects with small to large effects but demonstrated similar collecting of objects. ASD group also had significantly higher RSMS cluster scores, rates, and inventories of RRB w/ body; ASD group demonstrated more flapping-, rubbing-, patting-, and stiffening of body (S27).  **21.3m:** ASD group had significantly higher frequency and duration of observed RRB w/ objects. Specifically, ASD group displayed more banging/tapping-, rocking/flipping-, shaking-, swiping-, rubbing/squeezing-, spinning/wobbling-, rolling-, moving/placing-, and clutching objects with small to medium effect sizes but less lining up/stacking objects with a small effect size. ASD group displayed similar collecting of objects.  ASD group had significantly higher frequency and duration of observed RRB w/ body. Specifically, ASD group displayed more banging body against surface and patting-, rubbing-, and stiffening body with small to medium effect sizes but similar rocking/swiveling- and flapping body.  ASD group had significantly higher frequency and duration of observed sensory behaviors. Specifically, ASD group displayed more licking, feeling/touching, fixating, and sucking fingers with small effect sizes but displayed similar sniffing/smelling and covering of ears (S38).  **~24m:** ASD group had a significantly higher mean number of endorsed RSM items (ADI-R). Specifically, ASD group had significantly higher prevalence of and greater proportion of severe scores (scores of 2) on repetitive use of objects, complex mannerisms, hand/finger mannerisms, unusual preoccupations, and unusual attachments (S31).  **28.4m:** ASD group had significantly higher prevalence of hand/finger mannerisms, complex mannerisms, and repetitive behaviors (ADOS) at <18, 19-24, 25-30, 31-36, 37-42, and 43-56 months but  similar prevalence of stereotyped language (ADOS) (S18).  **41.3m:** Mean percent duration of observed motor, vocal, and total stereotypies during 10-minute behavior sample for 2 and 3-year-olds were significantly higher in the ASD group (S21).  **47.1m:** ASD group had significantly higher frequency and duration of observed repetitive behaviors overall. Specifically, (1) observed hand flapping was displayed by a significantly higher proportion of ASD group and at a significantly greater frequency and duration; (2) observed close gaze at objects was displayed by ASD group at a significantly greater frequency and duration, and (3) observed arm movements was displayed by a significantly greater proportion of ASD group.  ASD group had similar frequencies and durations of observed object exploration overall and also explored similar objects and similar numbers of objects.  Frequency and duration of observed repetitive behaviors overall and frequency and duration of observed object exploration overall were not correlated for ASD group but were positively correlated for TD group (S17). |
| **IS** | **12.6m:** At 12 months, HL-ASD group had similar RBS-R compulsive and ritualistic/sameness subscale inventory scores to HL-Neg group. At 24 months, HL-ASD group had significantly higher RBS-R ritualistic/sameness subscale inventory scores but similar RBS-R compulsive subscale inventory scores.  At 12 months, HL-ASD group had significantly higher RBS-R ritualistic/sameness subscale inventory scores than LL-Neg group but similar RBS-R compulsive subscale inventory scores. At 24 months, HL-ASD group had significantly higher inventory scores on both RBS-R subscales (S40).  **20.7m:** ASD group had similar scores on ritualized patterns of behavior and marked distress over change (SORF) (S9).  **~24m:** ASD group had a similar mean number of endorsed IS items (ADI-R). Specifically, ASD group had significantly higher prevalence of difficulties with changes in routine but similar prevalence of compulsions/rituals and resistance to trivial changes in the environment. ASD group had a significantly greater proportion of severe scores (scores of 2) on difficulty with changes in routine (S31). |
| **SIB** | **12.6m:** At 12 and 24 months, HL-ASD group had similar RBS-R SIB subscale inventory scores to HL-Neg.  At 12 months, HL-ASD group had similar RBS-R SIB subscale inventory scores to LL-Neg. At 24 months, HL-ASD group had significantly higher RBS-R SIB inventory scores (S40).  **~24m:** ASD group had similar prevalence of and proportion of severe scores (scores of 2) on SIB (ADI-R) (S31). |
| **Sensory** | **9-12m:** ASD group had similar rates of observed orienting and responding to auditory, visual, and tactile stimuli (S1).  **12.7m:** At 12 months, HL-ASD group had significantly higher SEQ hyper-responsivity and tactile modality scores than HL-Neg group but similar SEQ hypo-responsivity, sensory-seeking, auditory modality, and visual modality scores.  At 12 months, HL-ASD group had significantly higher SEQ hyper-responsivity scores than LL-Neg group but similar SEQ hypo-responsivity, sensory-seeking, auditory modality, visual modality, and tactile modality scores.  SEQ total, hypo-responsivity, and visual modality scores increased from 12 to 24 months for HL-ASD group but decreased for HL-Neg and LL-Neg groups. SEQ sensory-seeking scores decreased from 12 to 24 months for all groups. SEQ hyper-responsivity, auditory modality, and tactile modality scores did not change from 12 to 24 months for any group (S41).  **20.7m:** ASD group had similar scores on adverse response to sensory stimuli and unusual sensory exploration/interest (SORF) (S9).  **21.0m:** ASD group had similar scores for unusual sensory exploration of objects (SORF) (S39).  **~24m:** ASD group had significantly higher prevalence of unusual sensory interests and abnormal/idiosyncratic response to sensory stimuli but similar prevalence of sensitivity to noise (ADI-R). ASD group had a significantly greater proportion of severe scores (scores of 2) on unusual sensory interests and abnormal/idiosyncratic response to sensory stimuli (S31).  **24.7m:** HL-ASD group had significantly lower ITSP auditory processing scores and significantly lower scores in ITSP “low registration” reaction to sensory stimuli than HL-Neg and LL-Neg groups but similar ITSP visual-, tactile-, vestibular-, and oral sensory processing scores and similar scores in other reactions to sensory stimuli: sensory sensitivity, sensory-seeking, and sensory-avoiding (S11).  **28.4m:** ASD group had significantly higher prevalence of sensory interests (ADOS) at <18, 19-24, 25-30, 31-36, 37-42, and 43-56 months (S18).  **33.7m:** ASD group had significantly higher SSP total, taste/smell sensitivity, tactile sensitivity, and auditory filtering scores (S33).  **39.8m:** ASD group has significantly higher mean SEQ hypo-social, hyper-social, hypo-nonsocial, and hyper-nonsocial scores, controlling for maturational age (S3).  **43.8m**: ASD group had significantly higher SPA sensory aversion scores, controlling for chronological age but not when controlling for maturational age (S2). |
|  | **ASD vs. DD** |
| **Overall-RRB** | **20.7m:** ASD group had significantly higher ADOS-T RRB and SORF RRB scores (S9).  **21.0m:** At 20 and 42 months, ASD group had significantly higher ADI-R RRB scores but no specific RRB items differentiated ASD group from DD group (S7).  **21.0m:** ASD group had significantly higher SORF RRB scores (S39).  **~24m:** ASD group had a significantly higher mean number of endorsed ADI-R RRB items (S31).  **28.4m:** ASD group had significantly higher ADOS RRB scores and prevalence of ADOS RRB items at <18, 19-24, 25-30, 31-36, 37-42, and 43-56 months, controlling for age, MSEL NVIQ, and gender. Severity of ADOS RRB scores were stable over time for both ASD and DD groups (S18).  **33.7m:** ASD group had significantly higher ADI-R and ADOS-G RRB scores (S33).  **37.1m:** ASD group had significantly higher ADI-R RRB scores (S16). |
| **RSM** | **9-12m:** ASD group had significantly lower (more appropriate) observed object play ratings but similar rates of observed unusual posturing, repetitive leg and arm movements, and twiddling/waving-, banging/tapping-, mouthing-, staring at/fixating-, and rubbing objects (S1).  **12.0m:** ASD group displayed similar frequencies/durations of all observed typical object exploration behaviors: shaking/waving-, banging/tapping-, mouthing-, throwing/pushing object.  ASD group displayed significantly higher frequencies/duration of observed atypical object exploration behaviors: spinning-, rotating-, and unusual visual exploration of objects but a similar frequency of rolling objects (S29).  **20.7m:** ASD group had significantly higher scores on repetitive use of objects and excessive interest in particular objects but similar scores on repetitive body movements, actions, or activities, clutches particular objects, sticky attention to objects, fixation on parts of objects, and repetitive speech/intonation (SORF) (S9).  **21.0m:** ASD group had significantly higher scores for repetitive movements/posturing of body, arms, hands, or fingers and repetitive movements with objects but similar scores in lack of playing with a variety of toys conventionally and excessive interest in particular toys (SORF) (S39).  **21.3m:** ASD group had significantly higher RSMS composite scores.  ASD group had significantly higher RSMS cluster scores, rates and inventories of RRB w/ objects; specifically, ASD group demonstrated more swiping-, rubbing/squeezing-, rolling/knocking-, rocking/flipping-, spinning/wobbling-, moving/placing-, collecting-, and clutching objects with small to large effects but demonstrated similar lining up/stacking of objects.  ASD group also had significantly higher RSMS cluster scores and inventories (but not rates) of RRB w/ body; specifically, ASD group demonstrated more flapping-, rubbing-, and stiffening of body with small to medium effect sizes but demonstrated similar patting of body (S27).  **21.3m:** ASD group had significantly higher frequency and duration of observed RRB w/ objects. Specifically, ASD group displayed more banging/tapping-, rocking/flipping-, rolling-, shaking-, swiping-, spinning/wobbling-, collecting-, and clutching objects with small to medium effect sizes but less lining up/stacking objects with a medium effect size. ASD group displayed similar rubbing/squeezing- and moving/placing- objects.  ASD group had significantly higher frequency and duration of observed RRB w/ body. Specifically, ASD group displayed more banging body against surface, rubbing-, rocking/swiveling, and stiffening body with small to medium effect sizes but less flapping body with a small effect size. ASD group displayed similar patting body.  ASD group had significantly higher frequency and duration of observed sensory behaviors. Specifically, ASD group displayed more feeling/touching, fixating, and sucking fingers with small effect sizes but less licking- and sniffing/smelling body with small effect sizes. ASD group displayed similar covering of ears (S38).  **~24m:** ASD group had a significantly higher mean number of endorsed RSM items (ADI-R). Specifically, ASD group had significantly higher prevalence of repetitive use of objects, complex mannerisms, hand/finger mannerisms, unusual preoccupations, and unusual attachments. ASD group had a significantly greater proportion of severe scores (scores of 2) on all items but unusual attachments (S31).  **28.4m:** ASD group had significantly higher prevalence of hand/finger mannerisms, complex mannerisms, and repetitive behaviors (ADOS) at <18, 19-24, 25-30, 31-36, 37-42, and 43-56 months but similar prevalence of stereotyped language (ADOS) (S18). |
| **IS** | **20.7m:** ASD group had similar scores on ritualized patterns of behavior, and marked distress over change (SORF) (S9).  **~24m:** ASD group had a significantly higher mean number of endorsed IS items (ADI-R). ASD group had significantly higher prevalence of compulsions/rituals and difficulties with changes in routine but similar prevalence of resistance to trivial changes in the environment. ASD group had a significantly greater proportion of severe scores (scores of 2) on difficulty with changes in routine but not on compulsions/rituals (S31). |
| **SIB** | **~24m:** ASD group had similar prevalence of and proportion of severe scores (scores of 2) on SIB (ADI-R) (S31). |
| **Sensory** | **9-12m:** ASD group had similar rates of observed orienting and responding to auditory, visual, and tactile stimuli (S1).  **20.7m:** ASD group had similar scores on adverse response to sensory stimuli and unusual sensory exploration/interest (SORF) (S9).  **21.0m:** ASD group had similar scores for unusual sensory exploration of objects (SORF) (S39).  **~24m:** ASD group had significantly higher prevalence of unusual sensory interests and abnormal/idiosyncratic response to sensory stimuli but similar prevalence of sensitivity to noise (ADI-R). ASD group had a significantly greater proportion of severe scores (scores of 2) on unusual sensory interests and abnormal/idiosyncratic response to sensory stimuli (S31).  **28.4m:** ASD group had significantly higher prevalence of sensory interests (ADOS) at <18, 19-24, 25-30, 31-36, 37-42, and 43-56 months (S18).  **33.7m**: ASD group had significantly higher SSP total, taste/smell sensitivity, tactile sensitivity, and auditory filtering scores (S33).  **39.8m:** ASD group has significantly higher mean SEQ hypo-social and hypo-nonsocial scores but similar mean SEQ hyper-social and hyper-nonsocial scores, controlling for maturational age (S3).  **43.8m**: ASD group had similar SPA sensory aversion scores, controlling for either chronological or maturational age (S2). |

*Note*: Ages refer to mean age (months) of ASD sample (at intake/Time 1) within each study.

Groups: ASD = autism spectrum disorder (includes autism, autistic disorder, pervasive developmental disorder); DD = developmental delay; TD = typically developing; HL-ASD = higher-familial-likelihood-ASD; HL-Neg = higher-familial-likelihood-negative; LL-Neg = lower-familial-likelihood-negative.

Measures: ADI-R = Autism Diagnostic Interview - Revised; ADOS = Autism Diagnostic Observation Schedule; BISCUIT = Baby and Infant Scale for Children with aUtIsm Traits; CSBS = Communication and Symbolic Behavior Scales Developmental Profile; ITSP = Infant/Toddler Sensory Profile; MSEL = Mullen Scales of Early Learning; RBS-R = Repetitive Behavior Scale - Revised; RSMS = Repetitive and Stereotyped Movement Scales; SEQ = Sensory Experiences Questionnaire; SORF = Systematic Observation of Red Flags for Autism Spectrum Disorders in Young Children; SPA = Sensory Processing Assessment; SSP = Short Sensory Profile.
